# Effects of antioxidant nutrients on muscle mass, strength and function in COPD patients: A meta-analysis of randomized controlled trials

**DOI:** 10.1371/journal.pone.0316842

**Published:** 2025-01-17

**Authors:** Qinman He, Pan Yang, Ye Wang, Wanmei Xu, Yi Feng, Fei Xie, Guixiang Xu

**Affiliations:** Department of Respiratory and Critical Care Medicine, First Affiliated Hospital of Army Medical University, Chongqing, P. R. China; University of Turin: Universita degli Studi di Torino, ITALY

## Abstract

**Aim:**

To comprehensively investigate the effects of antioxidant nutrients on muscle mass, strength and function in chronic obstructive pulmonary disease (COPD) patients.

**Methods:**

PubMed, Embase, Cochrane Library, and Web of Science were comprehensively searched from the inception to January 3, 2024. The quality of randomized controlled trials (RCTs) was measured using the Jadad scale. Weighted mean differences (WMDs) and 95% confidence intervals (CIs) were used as the effect size for measurement data. Further, subgroup analysis was conducted based on whether patients participated in lung rehabilitation plans while receiving nutritional interventions. Sensitivity analysis was performed on all outcomes.

**Results:**

A total of 12 studies involving 595 patients with COPD were included, with 11 studies had high quality, and one study had low quality. For muscle mass, patients receiving antioxidant nutrients had a significantly increased lean body mass index compared with those not receiving antioxidant nutrients (pooled WMD: 0.903, 95% CI: 0.264, 1.541, *P* = 0.006). For patients who did not participate in lung rehabilitation plan while receiving nutritional interventions, antioxidant nutrients brought about a significantly higher lean body mass index (pooled WMD: 1.360, 95% CI: 0.560, 2.160, *P* = 0.001). For muscle strength, patients in the antioxidant nutrient intervention group had significantly higher hand grip strength (HGS) than those in the non-antioxidant nutrient intervention group (pooled WMD: 1.976, 95% CI: 1.337, 2.615, *P* < 0.001). Patients receiving antioxidant nutrients had significantly greater inspiratory muscle strength (MIP) than those not receiving antioxidant nutrients (pooled WMD: 8.127, 95% CI: 2.677, 13.577, *P* = 0.003).

**Conclusion:**

Antioxidant nutrient intervention significantly improved HGS, MIP and lean body mass index in COPD. Clinicians should consider increasing food intake or supplementation rich in antioxidants in the treatment plan of patients with COPD.

## Introduction

Chronic obstructive pulmonary disease (COPD) is a chronic inflammatory lung disorder feature by persistent respiratory symptoms and progressive airflow obstruction [1]. It is a primary cause of morbidity, mortality, and health-care use globally [2]. Malnutrition, a common problem in COPD, is a consequence of reduced nutritional intake and muscle loss, further exacerbated by systemic inflammation [3, 4], with a prevalence of around 25% in patients with COPD [5]. Malnourished individuals with COPD can have cachexia, sarcopenia and weight loss, poor exercise ability, muscle function and lung function as well as increased exacerbations, mortality and cost [6, 7]. Hence, providing nutritional support is necessary for COPD patients with malnutrition.

Nutritional support can improve energy and protein imbalances, leading to enhanced nutritional status and functional capacity [4, 8]. Several nutrients have been suggested to protect against airway destruction via antioxidant activity [9], and antioxidant nutrients are related to better lung function [10]. Antioxidant supplementation refers to the therapeutic approach of administering exogenous antioxidants to counteract oxidative stress, a state characterized by an imbalance between the production of reactive oxygen species (ROS) and the ability of the body to detoxify these reactive molecules [11]. Antioxidants, including but not limited to vitamins C and E, selenium, and beta-carotene, are substances that can delay or prevent cell damage caused by ROS [12]. Recently, whey beverage fortified with magnesium and vitamin C was shown to improve skeletal muscle mass and muscle strength, and further elevate health-related quality of life in patients with moderate-to-severe COPD [13]. Intravenous ferric carboxymaltose provided improvements in exercise capacity and functional restriction due to breathlessness in COPD [14]. In another randomized controlled trial (RCT), COPD patients exhibited significant improvements of muscle strength and other training-refractory outcomes after nutritional antioxidant supplementation [15]. At present, a meta-analysis by Collins et al. [16] found that nutritional support (food strategies, dietary advice, etc.), mainly in the form of oral nutritional supplements, improves total intake, anthropometric measures and grip strength in subjects with COPD. Ferreira et al. [17] showed in their review that nutritional supplementation (oral, enteral or parenteral nutritional support) increased expiratory muscle strength (MEP) and inspiratory muscle strength (MIP) among malnourished patients with COPD. As regards the role of antioxidant nutrients in COPD, few studies have been conducted. Lei et al. [18] demonstrated that vitamin C supplementation, a single antioxidant nutrient, could increase the levels of antioxidation in serum and improve lung function. However, the influences of comprehensive antioxidant nutrients in individuals with COPD remain unknown.

To fill this research gap, this meta-analysis of randomized controlled trials intended to comprehensively investigate the effects of antioxidant nutrients on muscle mass, strength and function in COPD patients, in order to provide a clinical reference for COPD management.

## Methods

This systematic review and meta-analysis was carried out following the Preferred Reporting Items for Systematic Reviews and Meta-analyses (PRISMA) [19]. The study was prospectively registered with the PROSPERO International Prospective Register of Systematic Reviews. The registration ID is CRD42024528396.

### Search strategy

PubMed, Embase, Cochrane Library, and Web of Science were comprehensively searched from the inception to January 3, 2024. Two investigators (QMH and PY) conducted the search independently. English search terms included the following: “Antioxidants” OR “Anti-Oxidants” OR “Anti Oxidants” OR “Antioxidant” OR “Anti-Oxidant” OR “Anti Oxidant” OR “Endogenous Antioxidants” OR “Antioxidants, Endogenous” OR “Endogenous Antioxidant” OR “Antioxidant, Endogenous” OR “Antioxidant Activity” OR “Activity, Antioxidant” OR “Antioxidant Effect” OR “Anti-Oxidant Effect” OR “Anti Oxidant Effect” OR “Anti-Oxidant Effects” OR “Anti Oxidant Effects” OR “Antioxidant Effects” OR “Whey Proteins” OR “Protein, Whey” OR “Proteins, Whey” OR “Whey Protein” AND “Pulmonary Disease, Chronic Obstructive” OR “Chronic Obstructive Lung Disease” OR “Chronic Obstructive Pulmonary Diseases” OR “COAD” OR “COPD” OR “Chronic Obstructive Airway Disease” OR “Chronic Obstructive Pulmonary Disease” OR “Airflow Obstruction, Chronic” OR “Airflow Obstructions, Chronic” OR “Chronic Airflow Obstructions” OR “Chronic Airflow Obstruction” OR “Bronchitis, Chronic” OR “Chronic Bronchitis” OR “Pulmonary Emphysema” OR “Emphysemas, Pulmonary” OR “Pulmonary Emphysemas” OR “Emphysema, Pulmonary” OR “Focal Emphysema” OR “Emphysema, Focal” OR “Emphysemas, Focal” OR “Focal Emphysemas” OR “Panacinar Emphysema” OR “Emphysema, Panacinar” OR “Emphysemas, Panacinar” OR “Panacinar Emphysemas” OR “Panlobular Emphysema” OR “Emphysema, Panlobular” OR “Emphysemas, Panlobular” OR “Panlobular Emphysemas” OR “Centriacinar Emphysema” OR “Centriacinar Emphysemas” OR “Emphysema, Centriacinar” OR “Emphysemas, Centriacinar” OR “Centrilobular Emphysema” OR “Centrilobular Emphysemas” OR “Emphysema, Centrilobular” OR “Emphysemas, Centrilobular”. The PubMed search strategy is shown in S1 File. The retrieved studies underwent a preliminary screening based on titles and abstracts, followed by screening through full text reading.

### Inclusion and exclusion criteria

The inclusion criteria were as follows: (1) studies on patients with COPD; (2) studies on patients receiving antioxidant nutrient intervention versus placebo or other therapies without antioxidant nutrient intervention, such as dietary recommendations and routine care; (3) studies on differences in muscle mass, strength, and function indicators (endpoint value minus baseline value); (4) RCTs; (5) English literature.

The exclusion criteria were as follows: (1) animal experiments; (2) retracted studies; (3) conference summaries, case reports, meta-analyses, reviews, editorial materials, letters, annotations, trial protocols, trial registration records; (4) studies without matched themes.

### Antioxidant nutrients and outcome measures

Our study incorporated a range of antioxidants supplements, including eicosapentaenoic acid (EPA)-enriched oral nutritional supplements, partially hydrolyzed whey protein, an oral mixture of essential amino acids, whey protein supplements, beverages fortified with magnesium and Vitamin C, omega-3 fatty acids, Vitamin D, Vitamin E, Vitamin C, zinc gluconate, selenium, and intravenous iron.

The outcome measures included muscle mass, strength, and function indicators. Muscle mass was evaluated by lean body mass, fat-free mass, lean body mass index, fat-free mass index, and skeletal muscle mass index. Muscle strength was assessed by hand grip strength (HGS), isometric maximal quadriceps strength (IMS Quad), MEP, and MIP. Muscle function was measured by six-minute walk distance (6MWD).

### Data extraction

Two investigators (QMH and PY) collected relevant data from the included studies, including author, year of publication, country, study design, group, way of treatment, sample size (N), male/female, age, body mass index (BMI), smoking, Global Initiative for Chronic Obstructive Lung Disease (GOLD) stages, pulmonary function, Jadad score, and outcomes.

### Quality assessment and risk of bias assessment

The quality of RCTs were measured using the Cochrane’s Risk of Bias tool [20], which comprises seven specified domains. Then, RCTs were classified into three categories: low risk, high risk, and having some concerns (S1A and S1B Fig).

### Quality of evidence assessment

The evidence quality underpinning each estimate was evaluated in accordance with the guidelines established by the Grading of Recommendations Assessment, Development and Evaluation (GRADE) Working Group [21]. This framework assesses the confidence in the evidence by considering elements such as methodological limitations of the studies, the degree of precision in the effect estimates, the presence of heterogeneity, the relevance of the evidence to the research question, and the potential for bias in the published literature, with a focus on the primary outcomes. The evidence is then classified into one of four quality levels: high, moderate, low, or very low (S2 File).

### Statistical analysis

All statistical analyses were performed with Stata 15.1 (Stata Corporation, College Station, TX, USA). Weighted mean differences (WMDs) and 95% confidence intervals (CIs) were used as the effect size for measurement data. The effect size of each outcome was tested for heterogeneity. If the heterogeneity statistic I^2^ ≥ 50%, the random-effects model was adopted for analysis; on the contrary, fixed-effects model was adopted. Further, subgroup analysis was conducted based on whether patients participated in lung rehabilitation plans while receiving nutritional interventions. Sensitivity analysis was performed on all outcomes. Publication biases were assessed via visual inspection of funnel plots for outcomes with 10 or more studies [22]. The difference was statistically significant if *P*<0.05.

## Results

### Characteristics of the included studies

**Fig 1:**
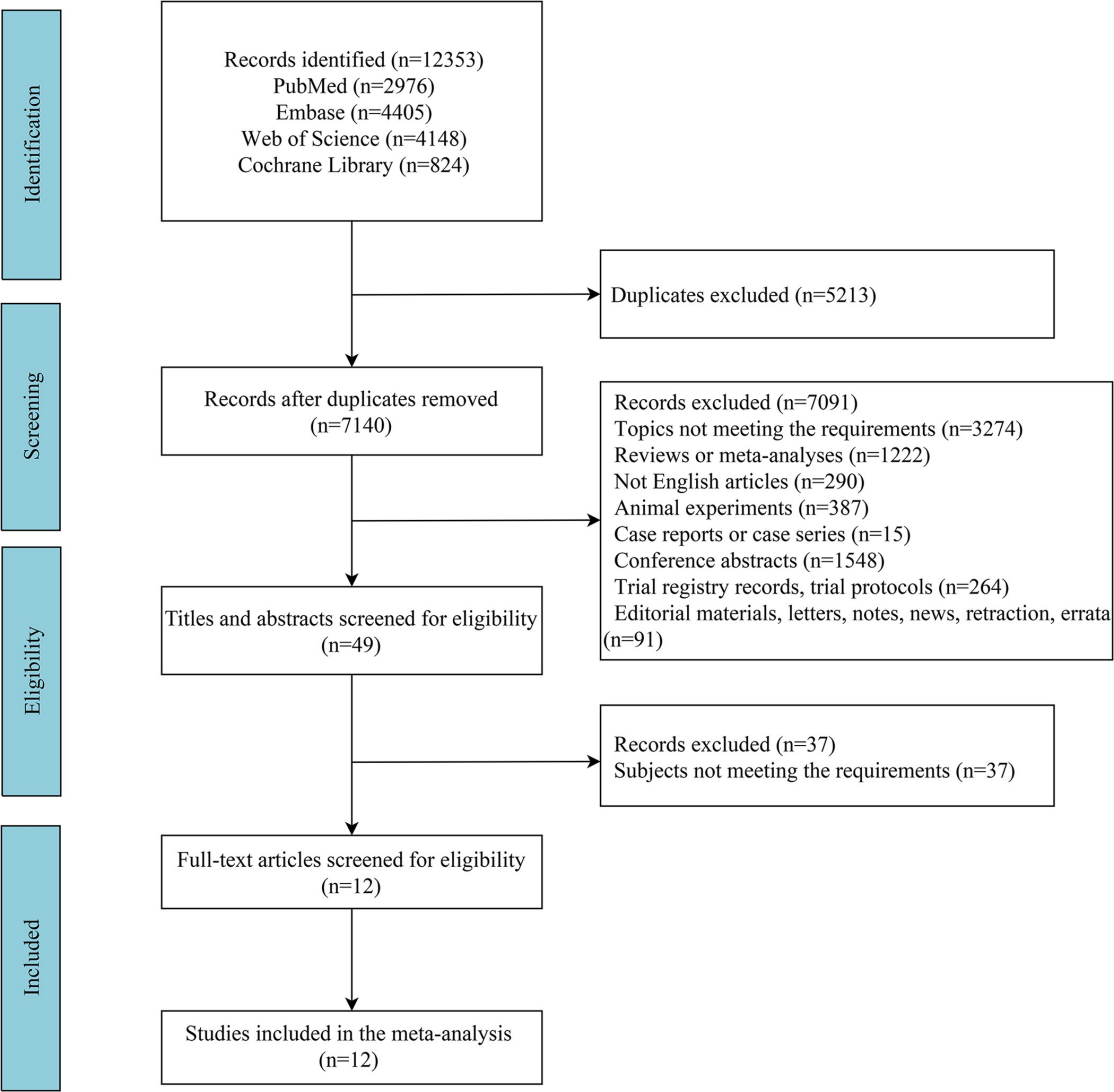
Selection process of eligible studies.

From the four databases, 12353 studies were retrieved. After duplicate removal, 7140 studies were left for screening based on titles and abstracts, and further on full texts. Finally, a total of 12 studies [13–15, 23–31] involving 595 patients with COPD were included in this analysis, with 296 patients in the antioxidant nutrient intervention group and 299 in the non-antioxidant nutrient intervention group. The selection process of eligible studies is shown in Fig 1. The year of publication ranged from 2007 to 2021. For the quality of the included studies, 11 studies had high quality, and one study had low quality. Table 1 illustrates the characteristics of the included studies.

**Table 1:** Characteristics of the included studies.

Author	Year	Country	Study design	Population	Group	Intervention type	Intervention Frequency	Intervention duration	Sample size	Male /Female	Age	BMI (kg/m2)	smoking	GOLD Stages	Pulmonary function	Outcome
Miek Hornikx	2012	Belgium	RCT	Moderate-to-severe COPD Patients	Intervention	Vitamin D	A monthly dose of 100.000 IU of vitamin D	3 months	25	19/6	67 ± 8 *	25 ± 5 *	-	Mild(I) 2 Moderate(II) 7 Severe(III) 12 Very severe(IV) 4	FEV1(L) 1.22 ± 0.50 * FVC (L) 2.96 ± 0.76 *	MEP,MIP,6MWD
					Control	Placebo			25	19/6	69 ± 6 *	24 ± 6 *	-	Mild(I) 0 Moderate(II) 6 Severe(III) 15 Very severe(IV) 4	FEV1(L) 1.06 ± 0.28 * FVC(L) 2.85 ± 0.80 *	
Rachida Rafiq	2017	Netherlands	RCT	COPD Patients who had a vitamin D deficiency (serum 25(OH)D<50 nmol/L)	Intervention	Vitamin D	1,200 IU colecalciferol during 6 months	6 months	24	13/11	64 [61–66] ^	29.6±6.7 *	Former 6 Current 18 Pack-years 33.5±20.2 *	I 6 II 8 III 8 IV 2	PEF (L) 4.75±2.46 * PEF (% predicted) 66.71±27.66 * FEV1 (L) 1.51[1.21–1.74] ^ FEV1 (% predicted) 58.46±21.23 * FVC (L) 3.37±0.89 * FVC (% predicted) 98.28±18.72 * FEV1/FVC 48.76±15.01 *	hand grip strength,MEP,MIP
					Control	Placebo			26	13/13	61 [58–66] ^	26.4±5.1 *	Former 8 Current 18 Pack-years 30.9±18.5 *	I 4 II 14 III 5 IV 3	PEF (L) 5.47±2.22 * PEF (% predicted) 73.95±24.71 * FEV1 (L) 1.67 [1.10–2.16] ^ FEV1 (% predicted) 58.95±23.03 * FVC (L) 3.52±1.16 * FVC (% predicted) 97.08±22.23 * FEV1/FVC 48.46±12.51 *	
Takashi Ogasawara	2018	Japan	RCT	patients with COPD and hospitalized for exacerbation of COPD or pneumonia	Intervention	EPA-enriched oral nutrition supplementation	1 g/day	Until discharge	24	21/3	77.4 ± 9.7 *	19.2 ± 2.5 *	-	I 6 II 12 III 5 IV 1	FVC (L) 2.62 ± 0.81 * FEV1 (L) 1.24 ± 0.56 * %FEV1 (%) 64.2 ± 24.7 *	skeletal muscle mass index,lean body mass,lean body mass index
					Control	EPA-free ONS of similar energy			21	20/1	79.1 ± 7.0 *	19.1 ± 2.8 *	-	I 5 II 9 III 4 IV 3	FVC (L) 2.51 ± 0.76 * FEV1 (L) 1.31 ± 0.68 * %FEV1 (%) 68.2 ± 34.8 *	
Claire de Bisschop	2021	France	RCT	Stable COPD patients	Intervention	BCAA supplementation	25g BCAA supplementation diluted in 150 mL water	4 weeks	25	7/18	65.4±8.8 *	-	Current smoker 8	-	FVC (L) 3.38±0.94 * FVC (% pred) 99.3±26.3 * FEV1 (L) 1.55 ±0.52 * FEV1 (% pred) 58.2 ±17.9 * FEV1/FVC (%) 47.8 ±16.8 *	left Isometric maximal quadriceps strength,right Isometric maximal quadriceps strength,6MWD
					Control	Placebo			29	11/18	64.4±8.0 *	-	Current smoker 5	-	FVC (L) 3.21±1.15 * FVC (% pred) 94.5±29.2 * FEV1 (L) 1.59 ±0.47 * FEV1 (% pred) 59.0 ±16.3 * FEV1/FVC (%) 51.2 ±12.2 *	
R.W. Dal Negro	2012	Verona	RCT	patients with stable, severe COPD	Intervention	Mixture of EAAs	4g of EAAs bid at 10:00 am and 5:00 pm	12 weeks	44	32/12	75±5 *	19.95±1.63 *	-	-	FEV1 (l/sec) 0.79±0.42 * FEV1/FVC (%) 39.49±7.47 *	fat-free mass,lean body mass index,hand grip strength
					Control	Undistinguishable dose of placebo			44	29/15	73±8 *	20.1±2 *	-	-	FEV1 (l/sec) 0.8±0.2 * FEV1/FVC (%) 37.7±11.59 *	
R.W. Dal Negro	2010	Verona	RCT	Patients with severe COPD and sarcopenia	Intervention	Mixture of EAAs	4 gr/bid EAAs	12 weeks	16	14/2	75±7 *	20.2±1.4 *	-	-	FEV1 (l/sec) 0.90±0.21 * FEV1/FVC (%) 39±7.18 *	fat-free mass,lean body mass index
					Control	Placebo			16	11/5	75±7 *	20.2±1.8 *	-	-	FEV1 (l/sec) 0.84±0.15 * FEV1/FVC (%) 38±11.5 *	
Christopher Lum	2007	Hong Kong	RCT	Elderly patients with COPD	Intervention	Whey protein	Sachets of whey protein supplement at 12 g twice daily	6weeks	25	23/2	76.1 (6.4) *	19.6 (3.9) *	-	-	FEV1 (absolute value, L /min) 0.48 (0.13) * % predicted FEV1 36.8% (20) *	
					Control	Placebo (identical looking sachets of casein)			24	22/2	71.3 (7.9) *	19.0 (3.8) *	-	-	FEV1 (absolute value, L /min) 0.48 (0.16) * % predicted FEV1 31.9% (15.1) *	
Afsane Ahmadi	2020	Iran	RCT	male patients with moderate-to-severe COPD	Intervention	Whey beverage fortified with magnesium and vitamin C	Daily received 250 ml of whey beverage fortified with magnesium and vitamin C	8 weeks	23		62.08 ± 7.0 *	20.65 ± 3.49 *	Smoking habit, yr. 32.73 ± 14.8 * Age starting smoking, yr. 26.36 ± 10.65 *	-	FEV1, % 42.58 ± 16.74 * FVC, % 59.29 ± 10.83 *	Lean body mass,Fat-free mass,lean body mass index,Right HGS,Left HGS
					Control	Dietary advice and routine care			23		63.47 ± 7.24 *	21.53 ± 2.59 *	Smoking habit, yr. 32.73 ± 14.8 * Age starting smoking, yr. 25.22 ± 11.55 *	-	FEV1, % 44.95 ± 14.16 * FVC, % 60.54 ± 10.34 *	
Philip C. Calder	2017	UK	RCT	Patients aged ≥50 years with moderate-to-severe COPD	Intervention	TMN (approximately 230 kcal; 10 g whey protein concentrate, minimum 2.0 g DHA + EPA, and 10 μg 25-hydroxy-vitamin D3 per 200 mL)	Drink two 200 mL study product containers daily	12 weeks	22	10/12	69.2 ± 6.3 *	22.5 ± 3.7 *	Current 13 Former 8	IV 15	FEV1 (% of FVC) 45.0 ± 10.0 *	Lean body mass,skeletal muscle mass index
					Control	Contained no 25-hydroxy-vitamin D3, milk protein instead of pure whey protein, and sunflower oil in place of omega-3 PUFA-containing fish oil (approximately 200 kcal per 200 mL)			23	13/10	69.7 ± 8.2 *	23.5 ± 4.0 *	Current 11 Former 11	IV 8	FEV1 (% of FVC) 52.4 ± 8.9 *	
Fares Gouzi	2019	France	RCT	Stable COPD patients (40 to 78 years old)	Intervention	Antioxidant supplements	α-tocopherol: 30 mg/day, ascorbate: 180 mg/day, zinc gluconate: 15 mg/day, selenomethionine: 50 μg/day	28 Days	31	15/16	62 4 ± 6 5 *	25 0 ± 4 2 *	pack years 45 ± 26 *	-	FEV1 (%pred) 57 ± 17 * FEV1/FVC ratio 41 ± 10 *	Muscle mass index, lean body mass index,6MWD
					Control	Placebo			26	13/13	61 1 ± 8 7 *	25 3 ± 4 7 *	pack years 40 ± 18 *	-	FEV1 (%pred) 62 ± 27 * FEV1/FVC ratio 43 ± 14 *	
Elham PIRABBASI	2016	Malaysia	RCT	male COPD patients,with moderate-to-severe COPD	Intervention	Vitamin C	Vitamin C (500 mg) once daily	6 Months	13		64.5 ± 10.2 *		pack years 26.8 ± 15.6 *	-		Lean body mass,lean body mass index
					Control	No intervention			18		64.17 ± 8.3 *		pack years 29.3 ± 24.4 *	-		
Peter Santer	2020	UK	RCT	Patients with moderate-to-severe COPD	Intervention	Ferric carboxymaltose	A single dose of intravenous ferric carboxymaltos (15mg/kg bodyweight)	1 week	24	15/9	69.2±8.4 *	25.7±6.1 *	Former 18 Current 6 Never 0 Pack-years 43 (31–67) ^	I - II 9 III 10 IV 5	FEV1, L 1.16±0.50 * FEV1, % of predicted 48.0±17.6 * FEV1/FVC, % 44.8±9.0 *	6MWD
					Control	Saline placebo			24	19/5	68.0±7.0 *	25.4±4.1 *	Former 16 Current 7 Never 1 Pack-years 39 (28–67) ^	I - II 10 III 10 IV 4	FEV1, L 1.35±0.38 * FEV1, % of predicted 49.8±16.9 * FEV1/FVC, % 440.4±10.2 *	

Notes: ^, Median [interquartile range]; *, mean ± SD; FeV1, forced expiratory volume in 1 second; FVC, forced vital capacity; SPPB, short physical performance battery; HGS, hand grip strength; MEP, expiratory muscle strength; MIP, inspiratory muscle strength; 6MWD, six-minute walk distance; GOLD, Global Initiative for Chronic Obstructive Lung Disease; ONS, oral nutrition supplementation; EPA, eicosapentaenoic acid; DHA, docosahexaenoic acid; PUFA, polyunsaturated fatty acid; BCAA, Branched-chain amino acids; EAA, essential amino acid.

### Muscle mass

#### Lean body mass

**Fig 2:**
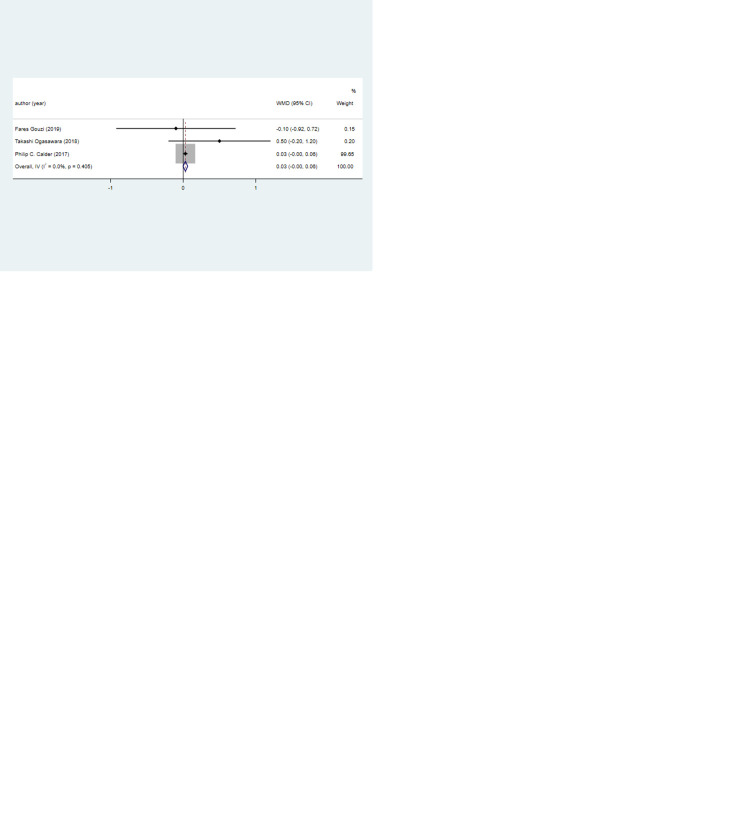
Forest plot for muscle mass in the antioxidant nutrient intervention group versus non-antioxidant nutrient intervention group. 2a, lean body mass; 2b, fat-free mass; 2c, lean body mass index; 2d, fat-free mass index; 2e, skeletal muscle mass index. WMD, weighted mean difference; CI, confidence interval.

The results of meta-analysis are shown in Table 2. Four studies with four sets of data assessed lean body mass. No significant difference was found in lean body mass between the antioxidant nutrient intervention and non-antioxidant nutrient intervention groups (pooled WMD: 0.404, 95% CI: -0.192, 1.000, *P* = 0.184). Regardless of whether patients participated in lung rehabilitation plans while receiving nutritional interventions, no significant association was observed between antioxidant nutrients and lean body mass (both *P* > 0.05) (Fig 2A).

**Table 2:** Pooled results of antioxidant nutrients for different outcomes.

Outcomes	Indicators	Number of studies	WMD (95%CI)	P	I^2^
Lean body mass	Nutritional intervention				
	Overall	4	0.404(-0.192,1.000)	0.184	66.0
	Subgroup analysis: whether patients participated in lung rehabilitation plans while receiving nutritional interventions				
	Yes	1	0.300(-3.666,4.266)	0.882	0.0
	No	3	0.413(-0.238,1.063)	0.214	77.3
	Moderate-to-severe COPD	3	0.413(-0.238,1.063)	0.214	77.3
Fat-free mass	Nutritional intervention				
	Overall	3	1.647(-0.882,4.176)	0.202	60.3
Lean body mass index	Nutritional intervention				
	Overall	3	0.903(0.264,1.541)	0.006	42.2
	Subgroup analysis: whether patients participated in lung rehabilitation plans while receiving nutritional interventions				
	Yes	1	0.100(-0.960,1.160)	0.853	0.0
	No	2	1.360(0.560,2.160)	0.001	0.0
	Moderate-to-severe COPD	2	1.360(0.560,2.160)	0.001	0.0
Fat-free mass index	Nutritional intervention				
	Overall	3	0.499(-0.158,1.157)	0.137	0.0
	Subgroup analysis: whether patients participated in lung rehabilitation plans while receiving nutritional interventions				
	Yes	1	0.000(-1.242,1.242)	1.000	0.0
	No	2	0.694(-0.081,1.469)	0.079	0.0
	Moderate-to-severe COPD	2	0.694(-0.081,1.469)	0.079	0.0
Skeletal muscle mass index	Nutritional intervention				
	Overall	3	0.031(-0.001,0.063)	0.057	0.0
	Subgroup analysis: whether patients participated in lung rehabilitation plans while receiving nutritional interventions				
	Yes	2	0.246(-0.288,0.781)	0.367	15.3
	No	1	0.030(-0.002,0.062)	0.064	0.0
HGS	Nutritional intervention				
	Overall	3	1.976(1.337,2.615)	<0.001	43.7
	Moderate-to-severe COPD	2	2.229(1.554,2.904)	<0.001	0.0
IMS quad	Nutritional intervention				
	Overall	1	0.869(-2.659,4.396)	0.629	74.0
MEP	Nutritional intervention				
	Overall	2	8.078(-5.251,21.407)	0.235	0.0
	Subgroup analysis: whether patients participated in lung rehabilitation plans while receiving nutritional interventions				
	Yes	1	7.000(-14.316,28.316)	0.520	0.0
	No	1	8.770(-8.309,25.849)	0.314	0.0
MIP	Nutritional intervention				
	Overall	2	8.127(2.677,13.577)	0.003	26.0
	Subgroup analysis: whether patients participated in lung rehabilitation plans while receiving nutritional interventions				
	Yes	1	11.000(3.709,18.291)	0.003	0.0
	No	1	4.490(-3.714,12.694)	0.283	0.0
6MWD	Nutritional intervention				
	Overall	5	3.489(-13.170,20.149)	0.681	27.1
	Subgroup analysis: whether patients participated in lung rehabilitation plans while receiving nutritional interventions				
	Yes	3	-2.954(-23.734,17.827)	0.781	55.1
	No	2	15.079(-12.792,42.950)	0.289	0.0
	Moderate-to-severe COPD	3	5.061(-28.314,38.435)	0.766	56.6

**Fat-free mass.** Fat-free mass was measured by three studies with three sets of data. The antioxidant nutrient intervention group had comparable fat-free mass to the non-antioxidant nutrient intervention group (pooled WMD: 1.647, 95% CI: -0.882, 4.176, *P* = 0.202) (Fig 2B).

#### Lean body mass index

The lean body mass index was evaluated by three studies with three sets of data. Patients receiving antioxidant nutrients had a significantly increased lean body mass index compared with those not receiving antioxidant nutrients (pooled WMD: 0.903, 95% CI: 0.264, 1.541, *P* = 0.006). For patients who did not participate in lung rehabilitation plan while receiving nutritional interventions, antioxidant nutrients brought about a significantly higher lean body mass index (pooled WMD: 1.360, 95% CI: 0.560, 2.160, *P* = 0.001) (Fig 2C).

#### Fat-free mass index

Three studies with three sets of data reported the fat-free mass index. There was no significant difference in the fat-free mass index between the antioxidant nutrient intervention and non-antioxidant nutrient intervention groups (pooled WMD: 0.499, 95% CI: -0.158, 1.157, *P* = 0.137). Regardless of whether patients participated in lung rehabilitation plans while receiving nutritional interventions, no significant association was found between antioxidant nutrients and the fat-free mass index (both *P* > 0.05) (Fig 2D).

#### Skeletal muscle mass index

The SMI was explored in three studies with three sets of data. The SMI of the antioxidant nutrient intervention group was similar to that of the non-antioxidant nutrient intervention group (pooled WMD: 0.031, 95% CI: -0.001, 0.063, *P* = 0.057). Regardless of whether patients participated in lung rehabilitation plans while receiving nutritional interventions, no significant association existed between antioxidant nutrients and the SMI (both *P* > 0.05) (Fig 2E).

### Muscle strength

#### HGS

**Fig 3:**
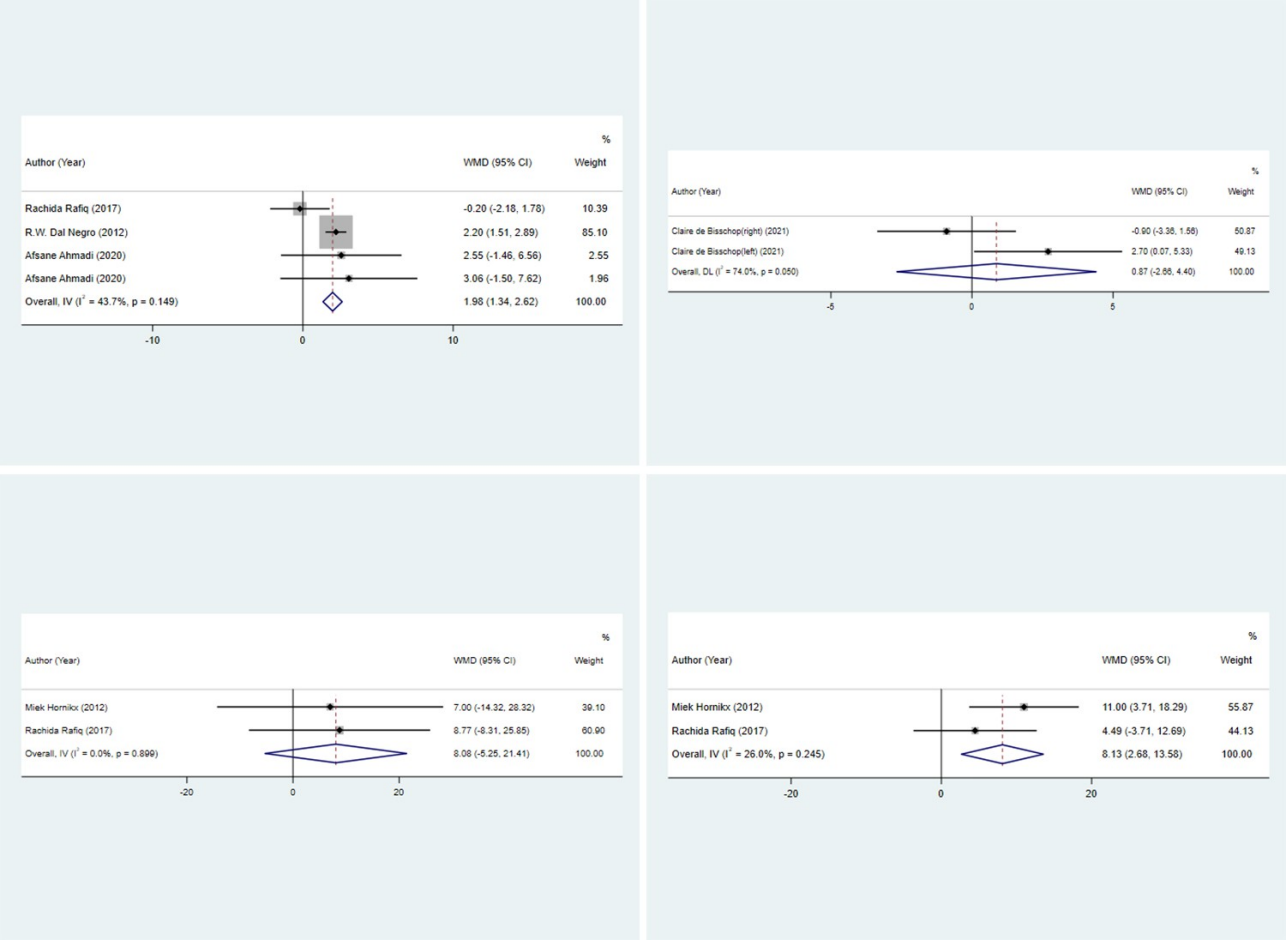
Forest plot for muscle strength in the antioxidant nutrient intervention group versus non-antioxidant nutrient intervention group. 3a, HGS; 3b, IMS Quad; 3c, MEP; 3d, MIP. HGS, hand grip strength; IMS Quad, isometric maximal quadriceps strength; MEP, expiratory muscle strength; MIP, inspiratory muscle strength; WMD, weighted mean difference; CI, confidence interval.

Three studies with four sets of data assessed HGS. Patients in the antioxidant nutrient intervention group had significantly higher HGS than those in the non-antioxidant nutrient intervention group (pooled WMD: 1.976, 95% CI: 1.337, 2.615, *P* < 0.001) (Fig 3A).

#### IMS Quad

IMS Quad was evaluated by one study with two sets of data. No significant difference was found in IMS Quad between the antioxidant nutrient intervention and non-antioxidant nutrient intervention groups (pooled WMD: 0.869, 95% CI: -2.659, 4.396, *P* = 0.629) (Fig 3B).

#### MEP

Two studies with two sets of data measured MEP. There was no significant difference in MEP between the antioxidant nutrient intervention and non-antioxidant nutrient intervention groups (pooled WMD: 8.078, 95% CI: -5.251, 21.407, *P* = 0.235). Regardless of whether patients participated in lung rehabilitation plans while receiving nutritional interventions, no significant association was observed between antioxidant nutrients and MEP (both *P* > 0.05) (Fig 3C).

#### MIP

Information on MIP was assessed by two studies with two sets of data. Patients receiving antioxidant nutrients had significantly greater MIP than those not receiving antioxidant nutrients (pooled WMD: 8.127, 95% CI: 2.677, 13.577, *P* = 0.003). For patients who participated in lung rehabilitation plan while receiving nutritional interventions, antioxidant nutrients resulted in significantly higher MIP (WMD: 11.000, 95% CI: 3.709, 18.291, *P* = 0.003) (Fig 3D).

### Muscle function

#### 6MWD

**Fig 4:**
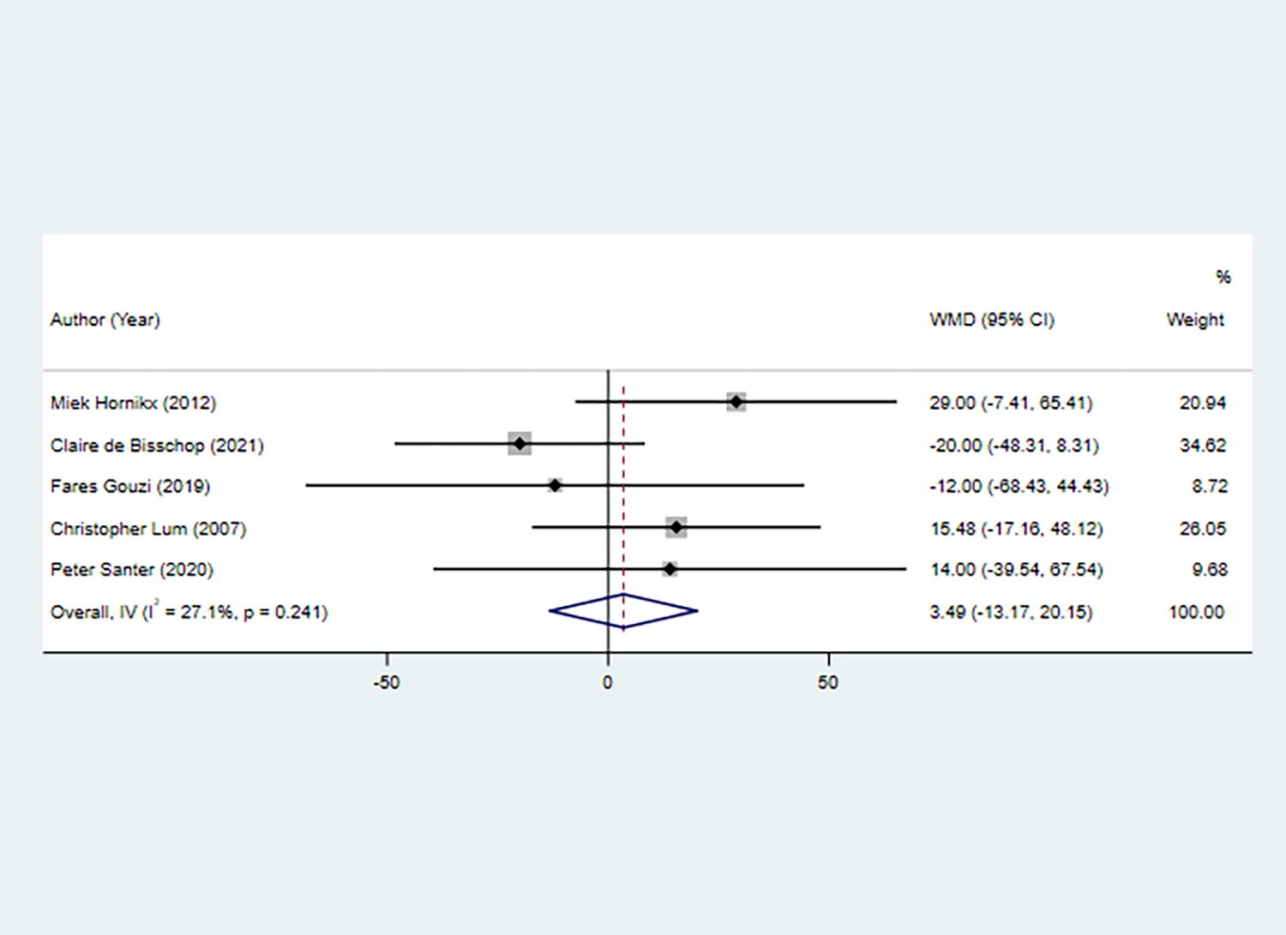
Forest plot for muscle function (6MWD) in the antioxidant nutrient intervention group versus non-antioxidant nutrient intervention group. 6MWD, six-minute walk distance; WMD, weighted mean difference; CI, confidence interval.

Five studies with five sets of data investigated the 6MWD. There was no significant difference in the 6MWD between the antioxidant nutrient intervention and non-antioxidant nutrient intervention groups (pooled WMD: 3.489, 95% CI: -13.170, 20.149, *P* = 0.681). Regardless of whether patients participated in lung rehabilitation plans while receiving nutritional interventions, no significant association was found between antioxidant nutrients and the 6MWD (both *P* > 0.05) (Fig 4).

### Sensitivity analysis

Sensitivity analysis was conducted by removing one study and synthetically analyzing the remaining studies. Its result exhibited that one-study removal did not significantly influence the overall outcomes, suggesting that the findings of this meta-analysis were stable and robust (S2–S4 Figs).

## Discussion

This meta-analysis probed into the effects of antioxidant nutrients on the muscle mass, strength and function of COPD patients, based on the 12 studies involving 595 patients. It was found that antioxidant nutrient intervention significantly improved HGS, MIP and lean body mass index in COPD; for patients who did not participate in lung rehabilitation plan while receiving nutritional interventions, antioxidant nutrients resulted in a significantly higher lean body mass index. Considering these findings, antioxidant nutrients may be used in clinical practice to increase the muscle mass and function of patients with COPD.

Several meta-analyses have explored the role of nutritional support in COPD. Collins et al. [8] reported that nutritional support in COPD led to significant improvements in a number of clinically relevant functional outcomes, while it was also demonstrated by another study that nutritional support had no influence on improving anthropometric measures, lung function, or functional exercise capacity in patients with stable COPD [32]. Ferreira et al. [17] showed in their review that nutritional supplementation was shown to enhance MEP and MIP in malnourished patients with COPD. Nevertheless, as regards the role of antioxidant nutrients in COPD, vitamin C supplementation, as found by Lei et al. [18], could increase the levels of antioxidation in serum and improve lung function. In a meta-analysis by Wang et al. [33], vitamin D supplementation improved the indicators of asthma and COPD, including pulmonary function and St. George’s Respiratory Questionnaire (SGRQ) scores. To make comprehensive analysis of various antioxidant nutrients in individuals with COPD, the current study was carried out, and COPD patients intervened with antioxidant nutrients had significantly elevated HGS, MIP and lean body mass index. Dietary intake of vitamin C and vitamin E are correlated with higher relative muscle strength according to the study of Shahinfar et al. [34]. The following may explain the positive influence of antioxidant nutrients on HGS, MIP and lean body mass index: (1) COPD patients exhibit heightened oxidative stress levels, with excessive production of free radicals leading to damage of pulmonary and systemic muscle tissues, including those responsible for respiratory function like inspiratory muscles [35–37]. Supplementation with antioxidants such as vitamin C and selenium can neutralize free radicals within the body, thus reducing oxidative stress-induced damage to muscle tissue and preserving and enhancing muscle strength [38]; (2) oxidative stress can lead to increased breakdown and decreased synthesis of muscle proteins, contributing to muscle atrophy [39]. Antioxidants protect muscle cells from oxidative stress-induced protein damage, helping to maintain a healthy balance between muscle protein synthesis and breakdown, thus preserving lean body mass [40]; (3) antioxidants can inhibit pro-inflammatory responses, improving the microenvironment within muscles to facilitate repair and regeneration of damaged muscle tissue [41]. Additionally, they can stimulate the release of muscle growth factors by activating certain signaling pathways, promoting muscle growth and recovery of strength [42]; (4) COPD patients often experience malnutrition, particularly protein-energy malnutrition [43]. Supplementation with antioxidants contributes to an overall improvement in nutritional status, enhancing immune function and metabolic processes, which in turn benefits muscle function improvement and weight maintenance [44]; (5) a high antioxidant state helps improve blood oxygen-carrying capacity and enhances energy metabolism within muscle cells [45], thereby increasing exercise tolerance and functional performance, including grip strength and inspiratory muscle strength. Notably, the mechanism underlying different antioxidant nutrients in COPD patients may vary depending on patient individual differences and the types, doses and treatment duration of nutrients, which necessitates clinical trials and individualized assessments.

In addition, among patients who did not participate in lung rehabilitation plan while receiving antioxidant nutrient interventions, antioxidant nutrients brought about a significantly higher lean body mass index. This finding suggested that clinicians can consider incorporating antioxidant nutrient supplementation into the standard treatment regimens for COPD patients, particularly for those not enrolled in pulmonary rehabilitation programs, as an effective means to enhance lean body mass and prevent muscle atrophy; and for patients who cannot attend pulmonary rehabilitation promptly or are unsuitable for such programs, improving lean body mass index through antioxidant nutrient supplementation serves as a simple and relatively cost-effective intervention, allowing for better utilization and allocation of healthcare resources. However, only two studies provided information on this subpopulation, which needs more studies for validation in the future.

The population included in the study included patients in the acute exacerbation and stable phases, with interventions including oral and injectable interventions. The analysis was not based on the final values of each outcome indicator, but on the difference relative to baseline, which better indicated that the changes were caused by the intervention. Given the positive role of antioxidant nutrients in COPD, clinical practitioners should consider increasing food intake or supplementation rich in antioxidants such as vitamin C, vitamin E, selenium, carotenoids, etc. in the treatment plan of COPD patients, as an auxiliary means of routine treatment. During the implementation of antioxidant nutrient intervention, regular monitoring of changes in patient HGS, MIP and lean body mass index is important indicators for evaluating treatment effectiveness, and nutritional intervention plans could be adjusted accordingly. Furthermore, education and counseling services on the benefits of antioxidant nutrients could be provided to COPD patients and their families, encouraging them to prioritize consumption of foods abundant in antioxidants in their daily lives and to use supplements judiciously under professional guidance when necessary.

There were some limitations. First, we acknowledge a limitation in our analysis related to the inability to perform subgroup analyses based on the type and frequency of antioxidant supplementation. The mixed composition of some supplements and the limited number of studies reporting on specific outcomes restricted our capacity to conduct these analyses. This limitation may affect the generalizability of our findings to specific supplement types or intake frequencies. Second, studies on muscle function could only be analyzed with 6MWD, while other methods such as the incremental cycling test and incremental shuttle walking test (ISWT) are evaluated by a small number of studies and cannot be used in this analysis. Third, the number of studies included for the outcome evaluation was relatively small, which may affect the reliability of the results. Fourth, there were limited number of studies available for outcomes. Consequently, we were unable to perform a comprehensive evaluation of publication bias using funnel plots, a common method that requires a sufficient number of studies to generate reliable results. This limitation could potentially affect the generalizability of our findings, as publication bias might influence the observed effect sizes and the overall conclusions drawn from the available literature. Fifth, one of the key limitations of this meta-analysis is the inability to extract data on daily energy and protein intake from the included studies. Many of the included studies did not report these essential variables, which are important factors influencing muscle mass, strength, and function. The absence of such data may limit our ability to fully assess the impact of antioxidant nutrients on muscle outcomes in the context of nutritional status. We acknowledge this gap in the data and recommend that future studies consider reporting energy and protein intake to provide a more comprehensive understanding of how these factors interact with antioxidant supplementation in COPD patients.

## Conclusion

Antioxidant nutrient intervention significantly improved HGS, MIP and lean body mass index in COPD; for patients who did not participate in lung rehabilitation plan while receiving antioxidant nutrients, antioxidant nutrients resulted in a significantly higher lean body mass index. Future studies are warranted to confirm these findings.

## Supporting information

S1 Fig Results of risk of bias. 1a, risk of bias graph; 1b, risk of bias summary.
(TIF)

S2 Fig Sensitivity analysis for muscle mass in the antioxidant nutrient intervention group versus non-antioxidant nutrient intervention group. 2a, lean body mass; 2b, fat-free mass; 2c, lean body mass index; 2d, fat-free mass index; 2e, skeletal muscle mass index.
(TIF)

S3 Fig Sensitivity analysis for muscle strength in the antioxidant nutrient intervention group versus non-antioxidant nutrient intervention group. 3a, HGS; 3b, IMS Quad; 3c, MEP; 3d, MIP.
(TIF)

S4 Fig Sensitivity analysis for muscle function (6MWD) in the antioxidant nutrient intervention group versus non-antioxidant nutrient intervention group. 6MWD, six-minute walk distance.
(TIF)

S1 File The PubMed search strategy. (DOCX)

S2 File The evidence quality assessed by the Grading of Recommendations Assessment, Development and Evaluation. (DOCX)

S3 File The PRISMA 2020 checklist. (DOCX)

S1 Raw data (ZIP)

## References

[1] Labaki WW, Rosenberg SR. Chronic Obstructive Pulmonary Disease. Ann Intern Med. 2020;173(3):Itc17–itc32. Epub 2020/08/04. doi: 10.7326/AITC202008040 . 32745458

[2] Christenson SA, Smith BM, Bafadhel M, Putcha N. Chronic obstructive pulmonary disease. Lancet. 2022;399(10342):2227–42. Epub 2022/05/10. doi: 10.1016/S0140-6736(22)00470-6 . 35533707

[3] Sehgal IS, Dhooria S, Agarwal R. Chronic obstructive pulmonary disease and malnutrition in developing countries. Curr Opin Pulm Med. 2017;23(2):139–48. Epub 2016/11/30. doi: 10.1097/MCP.0000000000000356 . 27898452

[4] Collins PF, Yang IA, Chang YC, Vaughan A. Nutritional support in chronic obstructive pulmonary disease (COPD): an evidence update. J Thorac Dis. 2019;11(Suppl 17):S2230–s7. Epub 2019/11/19. doi: 10.21037/jtd.2019.10.41 ; PubMed Central PMCID: PMC6831917. 31737350 PMC6831917

[5] Kaluźniak-Szymanowska A, Krzymińska-Siemaszko R, Deskur-Śmielecka E, Lewandowicz M, Kaczmarek B, Wieczorowska-Tobis K. Malnutrition, Sarcopenia, and Malnutrition-Sarcopenia Syndrome in Older Adults with COPD. Nutrients. 2021;14(1). Epub 2022/01/12. doi: 10.3390/nu14010044 ; PubMed Central PMCID: PMC8746722. 35010919 PMC8746722

[6] Keogh E, Mark Williams E. Managing malnutrition in COPD: A review. Respir Med. 2021;176:106248. Epub 2020/12/01. doi: 10.1016/j.rmed.2020.106248 . 33253970

[7] Hsieh MJ, Yang TM, Tsai YH. Nutritional supplementation in patients with chronic obstructive pulmonary disease. J Formos Med Assoc. 2016;115(8):595–601. Epub 2016/01/30. doi: 10.1016/j.jfma.2015.10.008 . 26822811

[8] Collins PF, Elia M, Stratton RJ. Nutritional support and functional capacity in chronic obstructive pulmonary disease: a systematic review and meta-analysis. Respirology. 2013;18(4):616–29. Epub 2013/02/26. doi: 10.1111/resp.12070 . 23432923

[9] Kim T, Choi H, Kim J. Association Between Dietary Nutrient Intake and Chronic Obstructive Pulmonary Disease Severity: A Nationwide Population-Based Representative Sample. Copd. 2020;17(1):49–58. Epub 2019/12/14. doi: 10.1080/15412555.2019.1698530 . 31833439

[10] Hu G, Cassano PA. Antioxidant nutrients and pulmonary function: the Third National Health and Nutrition Examination Survey (NHANES III). Am J Epidemiol. 2000;151(10):975–81. Epub 2000/06/15. doi: 10.1093/oxfordjournals.aje.a010141 . 10853636

[11] Mayne ST. Oxidative Stress, Dietary Antioxidant Supplements, and Health: Is the Glass Half Full or Half Empty? Cancer Epidemiology, Biomarkers & Prevention. 2013;22(12):2145–7. doi: 10.1158/1055-9965.EPI-13-1026 24130222

[12] Rafieian-Kopaei M, Baradaran A, Rafieian M. Plants antioxidants: From laboratory to clinic. J Nephropathol. 2013;2(2):152–3. Epub 2014/01/30. doi: 10.12860/JNP.2013.26 ; PubMed Central PMCID: PMC3891140. 24475444 PMC3891140

[13] Ahmadi A, Eftekhari MH, Mazloom Z, Masoompour M, Fararooei M, Eskandari MH, et al. Fortified whey beverage for improving muscle mass in chronic obstructive pulmonary disease: a single-blind, randomized clinical trial. Respir Res. 2020;21(1):216. Epub 2020/08/19. doi: 10.1186/s12931-020-01466-1 ; PubMed Central PMCID: PMC7430110. 32807165 PMC7430110

[14] Santer P, McGahey A, Frise MC, Petousi N, Talbot NP, Baskerville R, et al. Intravenous iron and chronic obstructive pulmonary disease: a randomised controlled trial. BMJ Open Respir Res. 2020;7(1). Epub 2020/06/23. doi: 10.1136/bmjresp-2020-000577 ; PubMed Central PMCID: PMC7311010. 32565444 PMC7311010

[15] Gouzi F, Maury J, Héraud N, Molinari N, Bertet H, Ayoub B, et al. Additional Effects of Nutritional Antioxidant Supplementation on Peripheral Muscle during Pulmonary Rehabilitation in COPD Patients: A Randomized Controlled Trial. Oxid Med Cell Longev. 2019;2019:5496346. Epub 2019/06/11. doi: 10.1155/2019/5496346 ; PubMed Central PMCID: PMC6501222. 31178967 PMC6501222

[16] Collins PF, Stratton RJ, Elia M. Nutritional support in chronic obstructive pulmonary disease: a systematic review and meta-analysis. Am J Clin Nutr. 2012;95(6):1385–95. Epub 2012/04/20. doi: 10.3945/ajcn.111.023499 . 22513295

[17] Ferreira IM, Brooks D, White J, Goldstein R. Nutritional supplementation for stable chronic obstructive pulmonary disease. Cochrane Database Syst Rev. 2012;12:Cd000998. Epub 2012/12/14. doi: 10.1002/14651858.CD000998.pub3 . 23235577 PMC11742366

[18] Lei T, Lu T, Yu H, Su X, Zhang C, Zhu L, et al. Efficacy of Vitamin C Supplementation on Chronic Obstructive Pulmonary Disease (COPD): A Systematic Review and Meta-Analysis. Int J Chron Obstruct Pulmon Dis. 2022;17:2201–16. Epub 2022/09/20. doi: 10.2147/COPD.S368645 ; PubMed Central PMCID: PMC9473551. 36118282 PMC9473551

[19] Page MJ, McKenzie JE, Bossuyt PM, Boutron I, Hoffmann TC, Mulrow CD, et al. The PRISMA 2020 statement: an updated guideline for reporting systematic reviews. Bmj. 2021;372:n71. Epub 2021/03/31. doi: 10.1136/bmj.n71 . 33782057 PMC8005924

[20] Higgins JP, Altman DG, Gøtzsche PC, Jüni P, Moher D, Oxman AD, et al. The Cochrane Collaboration’s tool for assessing risk of bias in randomised trials. Bmj. 2011;343:d5928. Epub 2011/10/20. doi: 10.1136/bmj.d5928 . 22008217 PMC3196245

[21] Puhan MA, Schünemann HJ, Murad MH, Li T, Brignardello-Petersen R, Singh JA, et al. A GRADE Working Group approach for rating the quality of treatment effect estimates from network meta-analysis. Bmj. 2014;349:g5630. Epub 2014/09/26. doi: 10.1136/bmj.g5630 . 25252733

[22] Sterne JA, Sutton AJ, Ioannidis JP, Terrin N, Jones DR, Lau J, et al. Recommendations for examining and interpreting funnel plot asymmetry in meta-analyses of randomised controlled trials. Bmj. 2011;343:d4002. Epub 2011/07/26. doi: 10.1136/bmj.d4002 . 21784880

[23] Rafiq R, Prins HJ, Boersma WG, Daniels JMA, den Heijer M, Lips P, et al. Effects of daily vitamin D supplementation on respiratory muscle strength and physical performance in vitamin D-deficient COPD patients: A pilot trial. International Journal of COPD. 2017;12:2583–92. doi: 10.2147/COPD.S132117 28894361 PMC5584776

[24] Pirabbasi E, Shahar S, Manaf ZA, Rajab NF, Manap RA. Efficacy of ascorbic acid (Vitamin C) and/N-acetylcysteine (NAC) supplementation on nutritional and antioxidant status of male chronic obstructive pulmonary disease (COPD) patients. Journal of Nutritional Science and Vitaminology. 2016;62(1):54–61. doi: 10.3177/jnsv.62.54 27117852

[25] Ogasawara T, Marui S, Miura E, Sugiura M, Matsuyama W, Aoshima Y, et al. Effect of eicosapentaenoic acid on prevention of lean body mass depletion in patients with exacerbation of chronic obstructive pulmonary disease: A prospective randomized controlled trial. Clin Nutr ESPEN. 2018;28:67–73. Epub 20181011. doi: 10.1016/j.clnesp.2018.09.076 . 30390895

[26] Lum C, Lo R, Ng K, Woo J, Tang N, Fallows S. A study on whey protein supplement on physical performance and quality of life among elderly patients with chronic obstructive pulmonary disease. Australasian Journal on Ageing. 2007;26(4):168–72. doi: 10.1111/j.1741-6612.2007.00257.x WOS:000251185200004.

[27] Hornikx M, Van Remoortel H, Lehouck A, Mathieu C, Maes K, Gayan-Ramirez G, et al. Vitamin D supplementation during rehabilitation in COPD: a secondary analysis of a randomized trial. Respiratory Research. 2012;13. doi: 10.1186/1465-9921-13-84 23006613 PMC3493348

[28] de Bisschop C, Caron F, Ingrand P, Bretonneau Q, Dupuy O, Meurice JC. Does branched-chain amino acid supplementation improve pulmonary rehabilitation effect in COPD? Respiratory Medicine. 2021;189. doi: 10.1016/j.rmed.2021.106642 34678585

[29] Dal Negro RW, Testa A, Aquilani R, Tognella S, Pasini E, Barbieri A, et al. Essential amino acid supplementation in patients with severe COPD: A step towards home rehabilitation. Monaldi Archives for Chest Disease—Pulmonary Series. 2012;77(2):67–75. doi: 10.4081/monaldi.2012.154 23193843

[30] Dal Negro RW, Aquilani R, Bertacco S, Boschi F, Micheletto C, Tognellai S. Comprehensive effects of supplemented essential amino acids in patients with severe COPD and sarcopenia. Monaldi Archives for Chest Disease—Pulmonary Series. 2010;73(1):25–33. doi: 10.4081/monaldi.2010.310 20499791

[31] Calder PC, Laviano A, Lonnqvist F, Muscaritoli M, Öhlander M, Schols A. Targeted medical nutrition for cachexia in chronic obstructive pulmonary disease: a randomized, controlled trial. Journal of Cachexia, Sarcopenia and Muscle. 2018;9(1):28–40. doi: 10.1002/jcsm.12228 28891198 PMC5803606

[32] Ferreira IM, Brooks D, Lacasse Y, Goldstein RS. Nutritional support for individuals with COPD: a meta-analysis. Chest. 2000;117(3):672–8. Epub 2000/03/14. doi: 10.1378/chest.117.3.672 . 10712990

[33] Wang Y, Wang J, Chen L, Zhang H, Yu L, Chi Y, et al. Efficacy of vitamin D supplementation on COPD and asthma control: A systematic review and meta-analysis. J Glob Health. 2022;12:04100. Epub 2022/12/16. doi: 10.7189/jogh.12.04100 . 36520525 PMC9754066

[34] Shahinfar H, Djafari F, Shahavandi M, Jalilpiran Y, Davarzani S, Clark CCT, et al. The lack of association between dietary antioxidant quality score with handgrip strength and handgrip endurance amongst Tehranian adults: A cross-sectional study from a Middle East country. Int J Clin Pract. 2021;75(4):e13876. Epub 2020/12/01. doi: 10.1111/ijcp.13876 . 33253498

[35] Loukides S, Bakakos P, Kostikas K. Oxidative stress in patients with COPD. Curr Drug Targets. 2011;12(4):469–77. Epub 2011/01/05. doi: 10.2174/138945011794751573 . 21194408

[36] Domej W, Oettl K, Renner W. Oxidative stress and free radicals in COPD—implications and relevance for treatment. Int J Chron Obstruct Pulmon Dis. 2014;9:1207–24. Epub 2014/11/08. doi: 10.2147/COPD.S51226 ; PubMed Central PMCID: PMC4207545. 25378921 PMC4207545

[37] Heunks LM, Dekhuijzen PN. Respiratory muscle function and free radicals: from cell to COPD. Thorax. 2000;55(8):704–16. Epub 2000/07/19. doi: 10.1136/thorax.55.8.704 ; PubMed Central PMCID: PMC1745815. 10899251 PMC1745815

[38] Barreiro E, de la Puente B, Minguella J, Corominas JM, Serrano S, Hussain SN, et al. Oxidative stress and respiratory muscle dysfunction in severe chronic obstructive pulmonary disease. Am J Respir Crit Care Med. 2005;171(10):1116–24. Epub 2005/03/01. doi: 10.1164/rccm.200407-887OC . 15735057

[39] Zhang H, Qi G, Wang K, Yang J, Shen Y, Yang X, et al. Oxidative stress: Roles in skeletal muscle atrophy. Biochem Pharmacol. 2023;214:115664. Epub 2023/06/19. doi: 10.1016/j.bcp.2023.115664 . 37331636

[40] Powers SK, Deminice R, Ozdemir M, Yoshihara T, Bomkamp MP, Hyatt H. Exercise-induced oxidative stress: Friend or foe? J Sport Health Sci. 2020;9(5):415–25. Epub 2020/05/08. doi: 10.1016/j.jshs.2020.04.001 ; PubMed Central PMCID: PMC7498668. 32380253 PMC7498668

[41] Parahuleva MS, Jung J, Burgazli M, Erdogan A, Parviz B, Hölschermann H. Vitamin C suppresses lipopolysaccharide-induced procoagulant response of human monocyte-derived macrophages. Eur Rev Med Pharmacol Sci. 2016;20(10):2174–82. Epub 2016/06/02. . 27249621

[42] Ji LL. Antioxidant signaling in skeletal muscle: a brief review. Exp Gerontol. 2007;42(7):582–93. Epub 2007/05/01. doi: 10.1016/j.exger.2007.03.002 . 17467943

[43] Nguyen HT, Collins PF, Pavey TG, Nguyen NV, Pham TD, Gallegos DL. Nutritional status, dietary intake, and health-related quality of life in outpatients with COPD. Int J Chron Obstruct Pulmon Dis. 2019;14:215–26. Epub 2019/01/23. doi: 10.2147/COPD.S181322 ; PubMed Central PMCID: PMC6336029. 30666102 PMC6336029

[44] Aubier M, Marthan R, Berger P, Chambellan A, Chanez P, Aguilaniu B, et al. [COPD and inflammation: statement from a French expert group: inflammation and remodelling mechanisms]. Rev Mal Respir. 2010;27(10):1254–66. Epub 2010/12/18. doi: 10.1016/j.rmr.2010.10.004 . 21163401

[45] Palmer AF, Intaglietta M. Blood substitutes. Annu Rev Biomed Eng. 2014;16:77–101. Epub 2014/05/14. doi: 10.1146/annurev-bioeng-071813-104950 . 24819476

