# Supplementary figures and images for: Effects of antioxidant nutrients on muscle mass, strength and function in COPD patients: A meta-analysis of randomized controlled trials
Source: PLoS One. 2025 Jan 17;20(1):e0316842. doi: 10.1371/journal.pone.0316842 (PMC11741611; doi:10.1371/journal.pone.0316842)

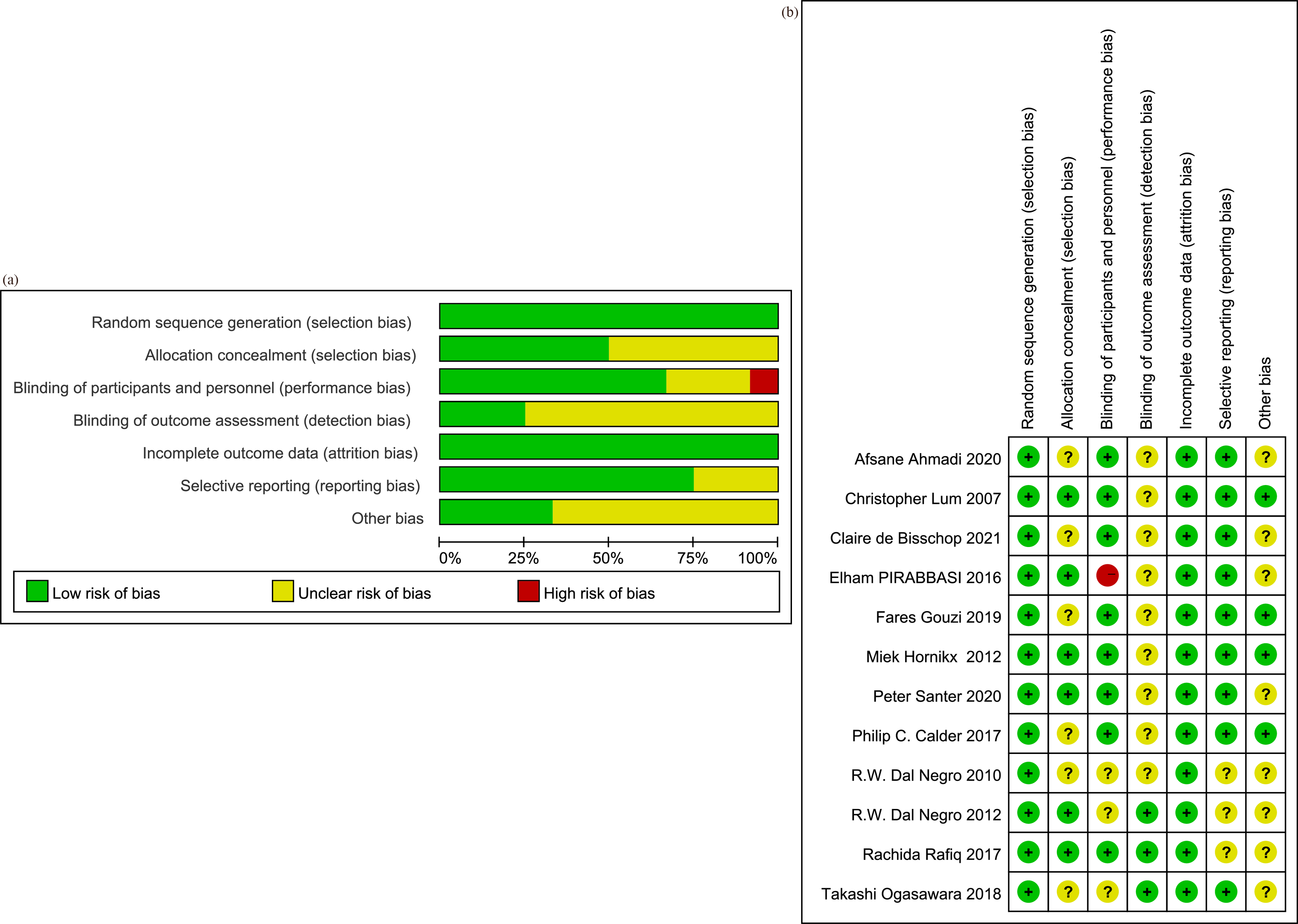

Supplement: S1 Fig — 1a, risk of bias graph; 1b, risk of bias summary. (TIF) [file pone.0316842.s001.tif]

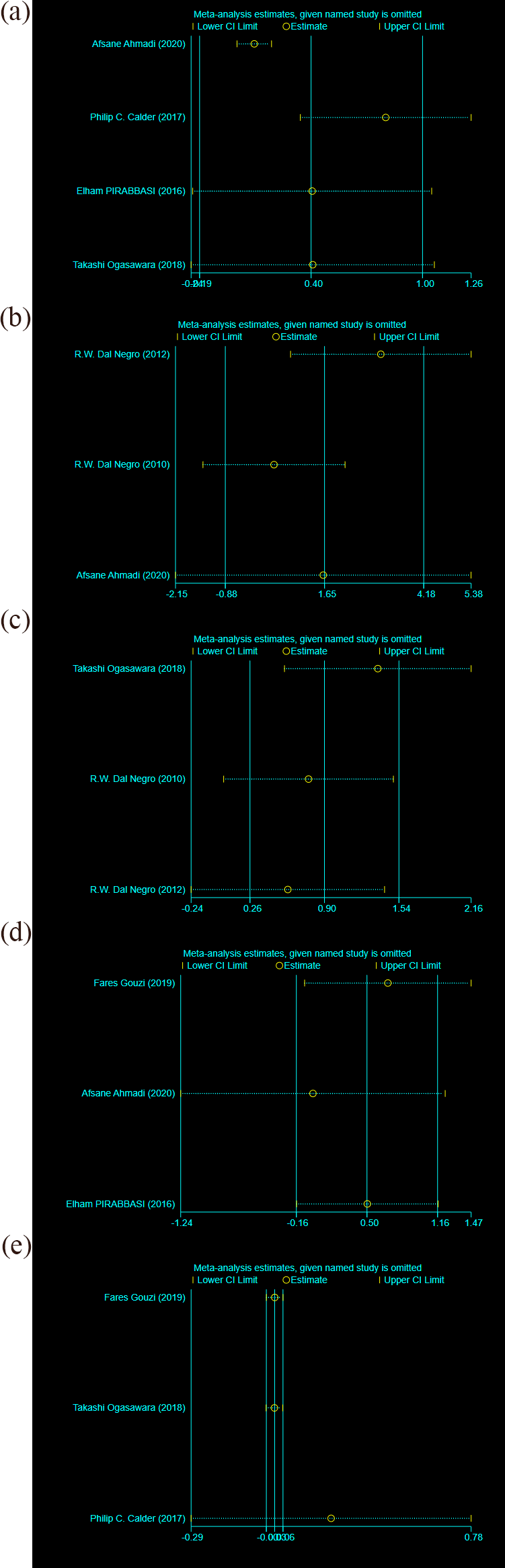

Supplement: S2 Fig — 2a, lean body mass; 2b, fat-free mass; 2c, lean body mass index; 2d, fat-free mass index; 2e, skeletal muscle mass index. (TIF) [file pone.0316842.s002.tif]

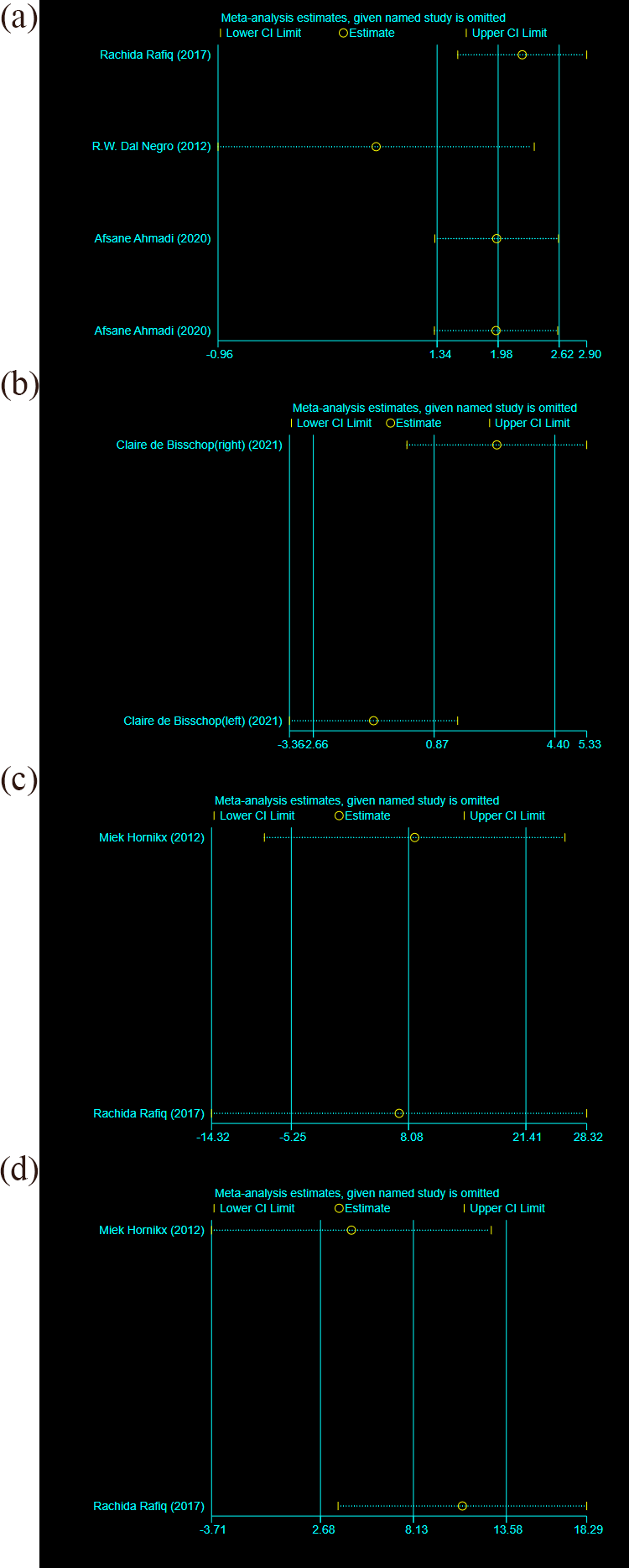

Supplement: S3 Fig — 3a, HGS; 3b, IMS Quad; 3c, MEP; 3d, MIP. (TIF) [file pone.0316842.s003.tif]

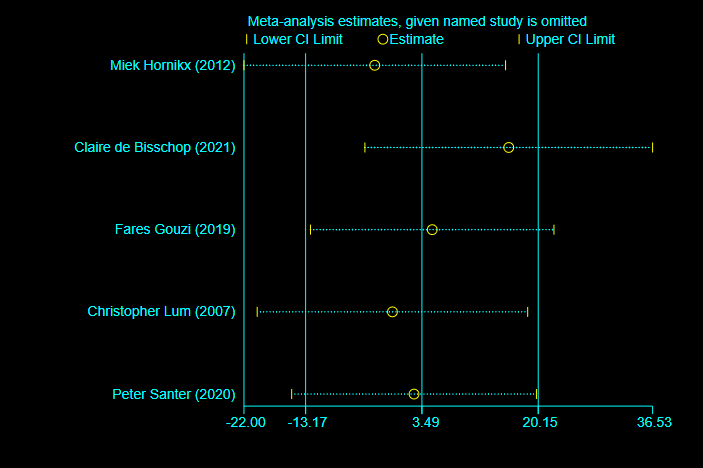

Supplement: S4 Fig — 6MWD, six-minute walk distance. (TIF) [file pone.0316842.s004.tif]

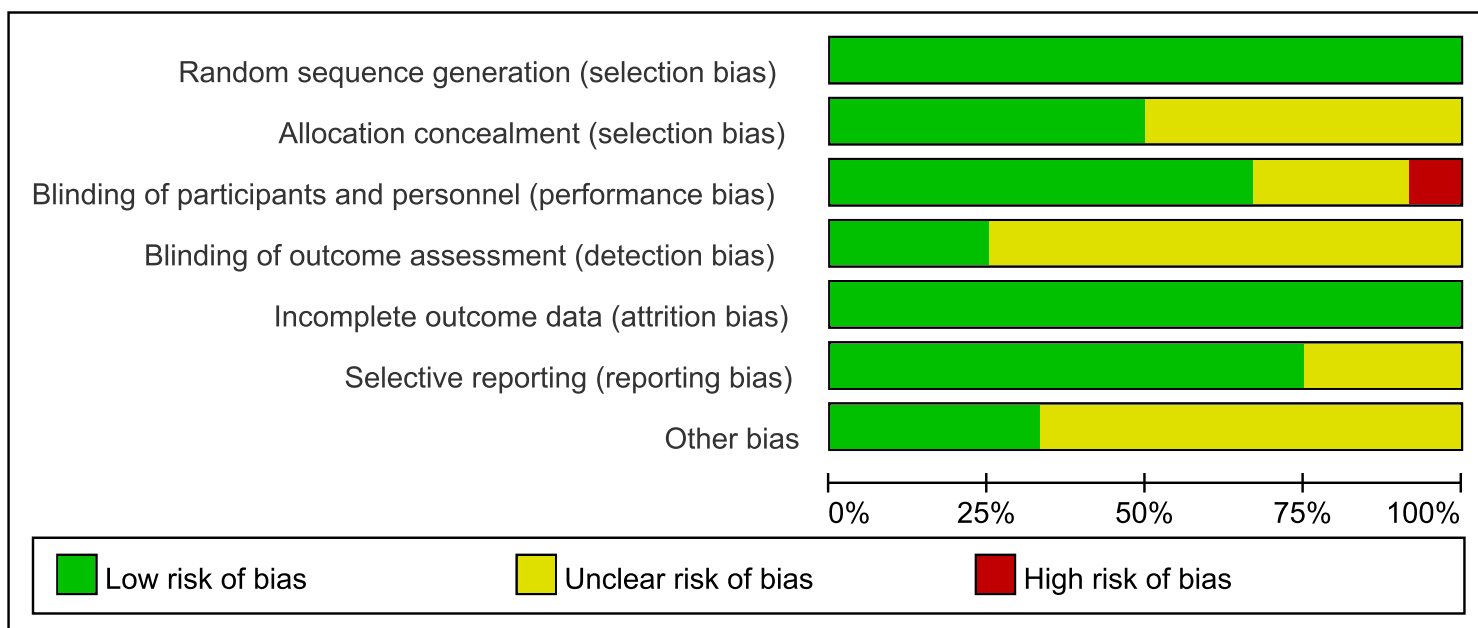

Supplement: S1 Raw data — (ZIP) [file pone.0316842.s008.zip › Raw data/Risk of bias graph.pdf]
