# Supplementary material for: Effects of antioxidant nutrients on muscle mass, strength and function in COPD patients: A meta-analysis of randomized controlled trials
Source: PLoS One. 2025 Jan 17;20(1):e0316842. doi: 10.1371/journal.pone.0316842 (PMC11741611; doi:10.1371/journal.pone.0316842)
Supplement: S1 File — (DOCX) [file pone.0316842.s005.docx]

((((((((((((((((((((((((((((((((((((((((((((((((((((Antioxidants[Title/Abstract]) OR (Anti-Oxidants[Title/Abstract])) OR (Anti Oxidants[Title/Abstract])) OR (Antioxidant[Title/Abstract])) OR (Anti-Oxidant[Title/Abstract])) OR (Anti Oxidant[Title/Abstract])) OR (Vitamin A[Title/Abstract])) OR (Aquasol A[Title/Abstract])) OR (Retinol[Title/Abstract])) OR (Ascorbic Acid[Title/Abstract])) OR (Acid, Ascorbic[Title/Abstract])) OR (L-Ascorbic Acid[Title/Abstract])) OR (Acid, L-Ascorbic[Title/Abstract])) OR (L Ascorbic Acid[Title/Abstract])) OR (Vitamin C[Title/Abstract])) OR (Hybrin[Title/Abstract])) OR (Magnorbin[Title/Abstract])) OR (Ascorbate[Title/Abstract])) OR (Ascorbicum[Title/Abstract])) OR (Vitamin D[Title/Abstract])) OR (Ergocalciferols[Title/Abstract])) OR (Calciferols[Title/Abstract])) OR (Vitamin D 2[Title/Abstract])) OR (Vitamin D2[Title/Abstract])) OR (D2, Vitamin[Title/Abstract])) OR (Ergocalciferol[Title/Abstract])) OR (Cholecalciferol[Title/Abstract])) OR (Calciol[Title/Abstract])) OR (Vitamin D 3[Title/Abstract])) OR (Vitamin D3[Title/Abstract])) OR (Cholecalciferols[Title/Abstract])) OR (Vitamin E[Title/Abstract])) OR (Tocopherol[Title/Abstract])) OR (beta Carotene[Title/Abstract])) OR (Carotene, beta[Title/Abstract])) OR (Betacarotene[Title/Abstract])) OR (beta-Carotene[Title/Abstract])) OR (Carotaben[Title/Abstract])) OR (Max-Caro[Title/Abstract])) OR (Max Caro[Title/Abstract])) OR (MaxCaro[Title/Abstract])) OR (Solatene[Title/Abstract])) OR (Vetoron[Title/Abstract])) OR (BellaCarotin[Title/Abstract])) OR (Provatene[Title/Abstract])) OR (Riboflavin[Title/Abstract])) OR (Vitamin G[Title/Abstract])) OR (Vitamin B2[Title/Abstract])) OR (Vitamin B 2[Title/Abstract])) OR (Nicotinamide[Title/Abstract])) OR (((((((((((((((((((((((((((((((((((((((((((((((((((((((Whey Proteins[Title/Abstract]) OR (Protein, Whey[Title/Abstract])) OR (Proteins, Whey[Title/Abstract])) OR (Whey Protein[Title/Abstract])) OR (Glutathione[Title/Abstract])) OR (gamma-L[Title/Abstract])) OR (gamma L[Title/Abstract])) OR (Glutathione[Title/Abstract])) OR (Cysteine[Title/Abstract])) OR (Cysteinate[Title/Abstract])) OR (Methionine[Title/Abstract])) OR (Pedameth[Title/Abstract])) OR (Liquimeth[Title/Abstract])) OR (Proline[Title/Abstract])) OR (Tryptophan[Title/Abstract])) OR (Levotryptophan[Title/Abstract])) OR (Ardeydorm[Title/Abstract])) OR (Ardeytropin[Title/Abstract])) OR (Trofan[Title/Abstract])) OR (Tryptacin[Title/Abstract])) OR (Tryptan[Title/Abstract])) OR (Optimax[Title/Abstract])) OR (Lyphan[Title/Abstract])) OR (Naturruhe[Title/Abstract])) OR (Fatty Acids, Omega-3[Title/Abstract])) OR (Omega-3 Fatty Acid[Title/Abstract])) OR (Acid, Omega-3 Fatty[Title/Abstract])) OR (Fatty Acid, Omega-3[Title/Abstract])) OR (Omega 3 Fatty Acid[Title/Abstract])) OR (Omega-3 Fatty Acids[Title/Abstract])) OR (n-3 Oil[Title/Abstract])) OR (Oil, n-3[Title/Abstract])) OR (n 3 Oil[Title/Abstract])) OR (n3 Oil[Title/Abstract])) OR (Oil, n3[Title/Abstract])) OR (n-3 Fatty Acids[Title/Abstract])) OR (n 3 Fatty Acids[Title/Abstract])) OR (Omega 3 Fatty Acids[Title/Abstract])) OR (n-3 PUFA[Title/Abstract])) OR (PUFA, n-3[Title/Abstract])) OR (n 3 PUFA[Title/Abstract])) OR (n3 Fatty Acid[Title/Abstract])) OR (Fatty Acid, n3[Title/Abstract])) OR (n3 PUFA[Title/Abstract])) OR (PUFA, n3[Title/Abstract])) OR (n3 Polyunsaturated Fatty Acid[Title/Abstract])) OR (n3 Oils[Title/Abstract])) OR (n-3 Oils[Title/Abstract])) OR (n 3 Oils[Title/Abstract])) OR (N-3 Fatty Acid[Title/Abstract])) OR (Acid, N-3 Fatty[Title/Abstract])) OR (Fatty Acid, N-3[Title/Abstract])) OR (N 3 Fatty Acid[Title/Abstract])) OR (n-3 Polyunsaturated Fatty Acid[Title/Abstract])) OR (n 3 Polyunsaturated Fatty Acid[Title/Abstract]))) OR ((((((((((((((((((((((((((((((((((((((((((((((((Eicosapentaenoic Acid[Title/Abstract]) OR (Eicosapentanoic Acid[Title/Abstract])) OR (Acid, Eicosapentanoic[Title/Abstract])) OR (Timnodonic Acid[Title/Abstract])) OR (Icosapent[Title/Abstract])) OR (Docosahexaenoic Acids[Title/Abstract])) OR (Acids, Docosahexaenoic[Title/Abstract])) OR (Docosahexenoic Acids[Title/Abstract])) OR (Acids, Docosahexenoic[Title/Abstract])) OR (Docosahexaenoic Acid[Title/Abstract])) OR (Acid, Docosahexaenoic[Title/Abstract])) OR (Docosahexaenoate[Title/Abstract])) OR (Fatty Acids, Omega-6[Title/Abstract])) OR (Acids, Omega-6 Fatty[Title/Abstract])) OR (Omega-6 Fatty Acid[Title/Abstract])) OR (Acid, Omega-6 Fatty[Title/Abstract])) OR (Fatty Acid, Omega-6[Title/Abstract])) OR (Omega 6 Fatty Acid[Title/Abstract])) OR (Omega-6 Fatty Acids[Title/Abstract])) OR (Omega 6 Fatty Acids[Title/Abstract])) OR (N-6 Fatty Acid[Title/Abstract])) OR (Acid, N-6 Fatty[Title/Abstract])) OR (Fatty Acid, N-6[Title/Abstract])) OR (N 6 Fatty Acid[Title/Abstract])) OR (Fatty Acids, Omega 6[Title/Abstract])) OR (N-6 Fatty Acids[Title/Abstract])) OR (Acids, N-6 Fatty[Title/Abstract])) OR (Fatty Acids, N-6[Title/Abstract])) OR (N 6 Fatty Acids[Title/Abstract])) OR (gamma-Linolenic Acid[Title/Abstract])) OR (Acid, gamma-Linolenic[Title/Abstract])) OR (gamma Linolenic Acid[Title/Abstract])) OR (Gamolenic Acid[Title/Abstract])) OR (Acid, Gamolenic[Title/Abstract])) OR (Linoleic Acids[Title/Abstract])) OR (Acids, Linoleic[Title/Abstract])) OR (Acids, Conjugated Linoleic[Title/Abstract])) OR (Linoleic Acid[Title/Abstract])) OR (Acid, Conjugated Linoleic[Title/Abstract])) OR (Linoleic Acid, Conjugated[Title/Abstract])) OR (Octadecadienoic[Title/Abstract])) OR (Linoleate[Title/Abstract])) OR (Linolelaidic Acid[Title/Abstract])) OR (Magnesium[Title/Abstract])) OR (Selenium[Title/Abstract])) OR (Zinc[Title/Abstract])) OR (Iron[Title/Abstract])) OR (Copper[Title/Abstract]))) AND ((((((((((((((Pulmonary Disease, Chronic Obstructive[Title/Abstract]) OR (Chronic Obstructive Lung Disease[Title/Abstract])) OR (Chronic Obstructive Pulmonary Diseases[Title/Abstract])) OR (COAD[Title/Abstract])) OR (COPD[Title/Abstract])) OR (Chronic Obstructive Airway Disease[Title/Abstract])) OR (Chronic Obstructive Pulmonary Disease[Title/Abstract])) OR (Airflow Obstruction, Chronic[Title/Abstract])) OR (Airflow Obstructions, Chronic[Title/Abstract])) OR (Chronic Airflow Obstructions[Title/Abstract])) OR (Chronic Airflow Obstruction[Title/Abstract])) OR ((((((((((((((((((((((((Pulmonary Emphysema[Title/Abstract]) OR (Emphysemas, Pulmonary[Title/Abstract])) OR (Pulmonary Emphysemas[Title/Abstract])) OR (Emphysema, Pulmonary[Title/Abstract])) OR (Focal Emphysema[Title/Abstract])) OR (Emphysema, Focal[Title/Abstract])) OR (Emphysemas, Focal[Title/Abstract])) OR (Focal Emphysemas[Title/Abstract])) OR (Panacinar Emphysema[Title/Abstract])) OR (Emphysema, Panacinar[Title/Abstract])) OR (Emphysemas, Panacinar[Title/Abstract])) OR (Panacinar Emphysemas[Title/Abstract])) OR (Panlobular Emphysema[Title/Abstract])) OR (Emphysema, Panlobular[Title/Abstract])) OR (Emphysemas, Panlobular[Title/Abstract])) OR (Panlobular Emphysemas[Title/Abstract])) OR (Centriacinar Emphysema[Title/Abstract])) OR (Centriacinar Emphysemas[Title/Abstract])) OR (Emphysema, Centriacinar[Title/Abstract])) OR (Emphysemas, Centriacinar[Title/Abstract])) OR (Centrilobular Emphysema[Title/Abstract])) OR (Centrilobular Emphysemas[Title/Abstract])) OR (Emphysema, Centrilobular[Title/Abstract])) OR (Emphysemas, Centrilobular[Title/Abstract]))) OR ((Bronchitis, Chronic[Title/Abstract]) OR (Chronic Bronchitis[Title/Abstract]))))
