# Supplementary material for: Effects of antioxidant nutrients on muscle mass, strength and function in COPD patients: A meta-analysis of randomized controlled trials
Source: PLoS One. 2025 Jan 17;20(1):e0316842. doi: 10.1371/journal.pone.0316842 (PMC11741611; doi:10.1371/journal.pone.0316842)
Supplement: S2 File — (DOCX) [file pone.0316842.s006.docx]

**Author(s):**

**Question:** Antioxidant nutrient compared to placebo or other therapies without antioxidant nutrient intervention for patients with COPD

**Setting:**

**Bibliography:**

| **Certainty assessment** | | | | | | | **№ of patients** | | **Effect** | | **Certainty** | **Importance** |
| --- | --- | --- | --- | --- | --- | --- | --- | --- | --- | --- | --- | --- |
| **№ of studies** | **Study design** | **Risk of bias** | **Inconsistency** | **Indirectness** | **Imprecision** | **Other considerations** | **antioxidant nutrient** | **placebo or other therapies without antioxidant nutrient intervention** | **Relative (95% CI)** | **Absolute (95% CI)** |  |  |
| **Hand grip strength** | | | | | | | | | | | | |
| 3 | Randomised trials | Not serious | Not serious | Serious^a^ | Not serious | None | 109 | 110 | - | MD **1.976 higher** (1.337 higher to 2.615 higher) | ⨁⨁⨁◯ Moderate |  |
| **Isometric maximal quadriceps strength** | | | | | | | | | | | | |
| 1 | Randomised trials | Not serious | Serious^b^ | Not serious | Serious^c^ | None | 48 | 58 | - | MD **0.869 higher** (2.659 lower to 4.396 higher) | ⨁⨁◯◯ Low |  |
| **Mep** | | | | | | | | | | | | |
| 2 | Randomised trials | Not serious | Not serious | Serious^d^ | Serious^c^ | None | 43 | 49 | - | MD **8.078 higher** (5.251 lower to 21.407 higher) | ⨁⨁◯◯ Low |  |
| **Mip** | | | | | | | | | | | | |
| 2 | Randomised trials | Not serious | Not serious | Serious^d^ | Serious^c^ | None | 43 | 49 | - | MD **8.127 higher** (2.677 higher to 13.577 higher) | ⨁⨁◯◯ Low |  |
| **6mwd** | | | | | | | | | | | | |
| 5 | Randomised trials | Not serious | Not serious | Not serious | Serious^c^ | None | 124 | 121 | - | MD **3.489 higher** (13.17 lower to 20.149 higher) | ⨁⨁⨁◯ Moderate |  |
| **Smi** | | | | | | | | | | | | |
| 3 | Randomised trials | Not serious | Not serious | Serious^a^ | Not serious | None | 74 | 66 | - | MD **0.031 higher** (0.001 lower to 0.063 higher) | ⨁⨁⨁◯ Moderate |  |
| **Fat-free mass** | | | | | | | | | | | | |
| 3 | Randomised trials | Not serious | Serious^b^ | Not serious | Serious^c^ | None | 83 | 81 | - | MD **1.647 higher** (0.882 lower to 4.176 higher) | ⨁⨁◯◯ Low |  |
| **Lean body mass** | | | | | | | | | | | | |
| 4 | Randomised trials | Serious^e^ | Not serious | Not serious | Not serious | None | 79 | 79 | - | MD **0.404 higher** (0.192 lower to 1 higher) | ⨁⨁⨁◯ Moderate |  |
| **Fat-free mass index** | | | | | | | | | | | | |
| 3 | Randomised trials | Serious^e^ | Not serious | Not serious | Not serious | None | 67 | 65 | - | MD **0.499 higher** (0.158 lower to 1.157 higher) | ⨁⨁⨁◯ Moderate |  |
| **Lean body mass index** | | | | | | | | | | | | |
| 3 | Randomised trials | Not serious | Not serious | Serious^a^ | Not serious | None | 84 | 81 | - | MD **0.903 higher** (0.264 higher to 1.541 higher) | ⨁⨁⨁◯ Moderate |  |

**CI:** confidence interval; **MD:** mean difference

#### Explanations

a. The severity of COPD varies among populations

b. High heterogeneity

c. 95% CI wide

d. Differences in comorbidities

e. Single-blind RCT
