# Supplementary material for: Effects of antioxidant nutrients on muscle mass, strength and function in COPD patients: A meta-analysis of randomized controlled trials
Source: PLoS One. 2025 Jan 17;20(1):e0316842. doi: 10.1371/journal.pone.0316842 (PMC11741611; doi:10.1371/journal.pone.0316842)
Supplement: S1 Raw data — (ZIP) [file pone.0316842.s008.zip › Raw data/Baseline information Raw data.docx]

| Author | Year | Country | study | Population | Group | Intervention type | Intervention Frequency | Intervention duration | N | Male  /Female | Age | BMI (kg/m2) | smoking | GOLD Stages | Pulmonary function | Outcome |
| --- | --- | --- | --- | --- | --- | --- | --- | --- | --- | --- | --- | --- | --- | --- | --- | --- |
| Miek Hornikx | 2012 | Belgium | RCT | moderate-to-severe COPD Patients | Intervention | vitamin D | a monthly dose of 100.000 IU of vitamin D | 3 Months | 25 | 19/6 | 67 ± 8 * | 25 ± 5 * | - | Mild(I) 2  Moderate(II) 7 Severe(III) 12 Very severe(IV) 4 | FEV1(L) 1.22 ± 0.50 * FVC (L) 2.96 ± 0.76 * | MEP,MIP,6MWD |
|  |  |  |  |  | Control | placebo |  |  | 25 | 19/6 | 69 ± 6 * | 24 ± 6 * | - | Mild(I) 0  Moderate(II) 6  Severe(III) 15 Very severe(IV) 4 | FEV1(L) 1.06 ± 0.28 * FVC(L) 2.85 ± 0.80 * |  |
| Rachida Rafiq | 2017 | Netherlands | RCT | COPD Patients who had a vitamin D deficiency (serum 25(OH)D<50 nmol/L) | Intervention | vitamin D | received 1,200 IU colecalciferol during 6 months | 6 months | 24 | 13/11 | 64 [61–66] ^ | 29.6±6.7 * | Former 6 Current 18 Pack-years 33.5±20.2 * | I 6 II 8 III 8 IV 2 | PEF (L) 4.75±2.46 * PEF (% predicted) 66.71±27.66 * FEV1 (L) 1.51[1.21–1.74] ^ FEV1 (% predicted) 58.46±21.23 * FVC (L) 3.37±0.89 * FVC (% predicted) 98.28±18.72 * FEV1/FVC 48.76±15.01 * | hand grip strength,MEP,MIP |
|  |  |  |  |  | Control | placebo |  |  | 26 | 13/13 | 61 [58–66] ^ | 26.4±5.1 * | Former 8 Current 18 Pack-years 30.9±18.5 * | I 4 II 14 III 5 IV 3 | PEF (L) 5.47±2.22 * PEF (% predicted) 73.95±24.71 * FEV1 (L) 1.67 [1.10–2.16] ^ FEV1 (% predicted) 58.95±23.03 * FVC (L) 3.52±1.16 * FVC (% predicted) 97.08±22.23 * FEV1/FVC 48.46±12.51 * |  |
| Takashi Ogasawara | 2018 | Japan | RCT | patients with COPD and hospitalized for exacerbation of COPD or pneumonia | Intervention | EPA-enriched oral nutrition supplementation | 1 g/day | Until discharge | 24 | 21/3 | 77.4 ± 9.7 * | 19.2 ± 2.5 * | - | I 6 II 12 III 5 IV 1 | FVC (L) 2.62 ± 0.81 * FEV1 (L) 1.24 ± 0.56 * %FEV1 (%) 64.2 ± 24.7 * | skeletal muscle mass index,lean body mass,lean body mass index |
|  |  |  |  |  | control | EPA-free ONS of similar energy |  |  | 21 | 20/1 | 79.1 ± 7.0 * | 19.1 ± 2.8 * | - | I 5 II 9 III 4 IV 3 | FVC (L) 2.51 ± 0.76 * FEV1 (L) 1.31 ± 0.68 * %FEV1 (%) 68.2 ± 34.8 * |  |
| Claire de Bisschop | 2021 | France | RCT | Stable COPD patients | Intervention | BCAA supplementation | 25g BCAA supplementation diluted in 150 mL water | 4 weeks | 25 | 7/18 | 65.4±8.8 * | - | Current smoker 8 | - | FVC (L) 3.38±0.94 * FVC (% pred) 99.3±26.3 * FEV1 (L) 1.55 ±0.52 * FEV1 (% pred) 58.2 ±17.9 * FEV1/FVC (%) 47.8 ±16.8 * | left Isometric maximal quadriceps strength,right Isometric maximal quadriceps strength,6MWD |
|  |  |  |  |  | control | placebo |  |  | 29 | 11/18 | 64.4±8.0 * | - | Current smoker 5 | - | FVC (L) 3.21±1.15 * FVC (% pred) 94.5±29.2 * FEV1 (L) 1.59 ±0.47 * FEV1 (% pred) 59.0 ±16.3 * FEV1/FVC (%) 51.2 ±12.2 * |  |
| R.W. Dal Negro | 2012 | Verona | RCT | patients with stable, severe COPD | Intervention | mixture of EAAs | 4g of EAAs bid at 10:00 am and 5:00 pm | 12 weeks | 44 | 32/12 | 75±5 * | 19.95±1.63 * | - | - | FEV1 (l/sec) 0.79±0.42 * FEV1/FVC (%) 39.49±7.47 * | fat-free mass,lean body mass index,hand grip strength |
|  |  |  |  |  | control | undistinguishable dose of placebo |  |  | 44 | 29/15 | 73±8 * | 20.1±2 * | - | - | FEV1 (l/sec) 0.8±0.2 * FEV1/FVC (%) 37.7±11.59 * |  |
| R.W. Dal Negro | 2010 | Verona | RCT | Patients with severe COPD and sarcopenia | Intervention | mixture of EAAs | 4 gr/bid EAAs | 12 weeks | 16 | 14/2 | 75±7 * | 20.2±1.4 * | - | - | FEV1 (l/sec) 0.90±0.21 * FEV1/FVC (%) 39±7.18 * | fat-free mass,lean body mass index |
|  |  |  |  |  | control | placebo |  |  | 16 | 11/5 | 75±7 * | 20.2±1.8 * | - | - | FEV1 (l/sec) 0.84±0.15 *  FEV1/FVC (%) 38±11.5 * |  |
| Christopher Lum | 2007 | Hong Kong | RCT | Elderly patients with COPD | Intervention | whey protein | sachets of whey protein supplement at 12 g twice daily | 6weeks | 25 | 23/2 | 76.1 (6.4) * | 19.6 (3.9) * | - | - | FEV1 (absolute value, L /min) 0.48 (0.13) * % predicted FEV1 36.8% (20) * |  |
|  |  |  |  |  | control | placebo(identical looking sachets of casein) |  |  | 24 | 22/2 | 71.3 (7.9) * | 19.0 (3.8) * | - | - | FEV1 (absolute value, L /min) 0.48 (0.16) * % predicted FEV1 31.9% (15.1) * |  |
| Afsane Ahmadi | 2020 | Iran | RCT | male patients with moderate-to-severe COPD | Intervention | whey beverage fortified with magnesium and vitamin C | daily received 250 ml of whey beverage fortified with magnesium and vitamin C | 8 weeks | 23 |  | 62.08 ± 7.0 * | 20.65 ± 3.49 * | Smoking habit, yr. 32.73 ± 14.8 * Age starting smoking, yr.  26.36 ± 10.65 * | - | FEV1, % 42.58 ± 16.74 * FVC, % 59.29 ± 10.83 * | Lean body mass,Fat-free mass,lean body mass index,Right HGS,Left HGS |
|  |  |  |  |  | control | dietary advice and routine care |  |  | 23 |  | 63.47 ± 7.24 * | 21.53 ± 2.59 * | Smoking habit, yr. 32.73 ± 14.8 * Age starting smoking, yr.  25.22 ± 11.55 * | - | FEV1, % 44.95 ± 14.16 * FVC, % 60.54 ± 10.34 * |  |
| Philip C. Calder | 2017 | UK | RCT | Patients aged ≥50 years with moderate-to-severe COPD | Intervention | TMN (approximately 230 kcal; 10 g whey protein concentrate, minimum 2.0 g DHA + EPA, and 10 μg 25-hydroxy-vitamin D3 per 200 mL) | drink two 200 mL study product containers daily | 12 weeks | 22 | 10/12 | 69.2 ± 6.3 * | 22.5 ± 3.7 * | Current 13  Former 8 | IV 15 | FEV1 (% of FVC) 45.0 ± 10.0 * | Lean body mass,skeletal muscle mass index |
|  |  |  |  |  | control | contained no 25-hydroxy-vitamin D3, milk protein instead of pure whey protein, and sunflower oil in place of omega-3 PUFA-containing fish oil (approximately 200 kcal per 200 mL) |  |  | 23 | 13/10 | 69.7 ± 8.2 * | 23.5 ± 4.0 * | Current 11  Former 11 | IV 8 | FEV1 (% of FVC) 52.4 ± 8.9 * |  |
| Fares Gouzi | 2019 | France | RCT | Stable COPD patients (40 to 78 years old) | Intervention | Antioxidant supplements | α-tocopherol: 30 mg/day, ascorbate: 180 mg/day, zinc gluconate: 15 mg/day, selenomethionine: 50 μg/day | 28 Days | 31 | 15/16 | 62 4 ± 6 5 * | 25 0 ± 4 2 * | pack years 45 ± 26 * | - | FEV1 (%pred) 57 ± 17 * FEV1/FVC ratio 41 ± 10 * | Muscle mass index ,lean body mass index,6MWD |
|  |  |  |  |  | control | placebo |  |  | 26 | 13/13 | 61 1 ± 8 7 * | 25 3 ± 4 7 * | pack years 40 ± 18 * | - | FEV1 (%pred) 62 ± 27 * FEV1/FVC ratio 43 ± 14 * |  |
| Elham PIRABBASI | 2016 | Malaysia | RCT | male COPD patients,with moderate-to-severe COPD | Intervention | Vitamin C | vitamin C (500 mg) once daily | 6 Months | 13 |  | 64.5 ± 10.2 * |  | pack years  26.8 ± 15.6 * | - |  | Lean body mass,lean body mass index |
|  |  |  |  |  | control | No intervention |  |  | 18 |  | 64.17 ± 8.3 * |  | pack years  29.3 ± 24.4 * | - |  |  |
| Peter Santer | 2020 | UK | RCT | Patients with moderate-to-severe COPD | Intervention | ferric carboxymaltose | a single dose of intravenous ferric carboxymaltos(15mg/kg bodyweight) | 1 week | 24 | 15/9 | 69.2±8.4 * | 25.7±6.1 * | Former 18 Current 6 Never 0 Pack-years  43 (31–67) ^ | I - II 9 III 10 IV 5 | FEV1, L 1.16±0.50 * FEV1, % of predicted 48.0±17.6 * FEV1/FVC, % 44.8±9.0 * | 6MWD |
|  |  |  |  |  | control | saline placebo |  |  | 24 | 19/5 | 68.0±7.0 * | 25.4±4.1 * | Former 16 Current 7 Never 1 Pack-years  39 (28–67) ^ | I - II 10 III 10 IV 4 | FEV1, L 1.35±0.38 * FEV1, % of predicted 49.8±16.9 * FEV1/FVC, % 440.4±10.2 * |  |

注：^,median [interquartile range];*,mean ± SD;COPD,chronic obstructive pulmonary disease;FeV1,forced expiratory volume in 1 second; FVC, forced vital capacity; SPPB,short physical performance battery;MIP,Inspiratory Muscle Strength;MEP,Expiratory Muscle Strength;6MWD,Six Minutes Walking Distance;GOLD,Global Initiative for Chronic Obstructive Lung Disease;ONS,oral nutrition supplementation;EPA,eicosapentaenoic acid；DHA，docosahexaenoic acid;PUFA,polyunsaturated fatty acid;BCAA,Branched-chain amino acids;EAA,essential amino acid;BCAA,Branched-chain amino acid
