# Supplementary material for: Effects of antioxidant nutrients on muscle mass, strength and function in COPD patients: A meta-analysis of randomized controlled trials
Source: PLoS One. 2025 Jan 17;20(1):e0316842. doi: 10.1371/journal.pone.0316842 (PMC11741611; doi:10.1371/journal.pone.0316842)
Supplement: S1 Raw data — (ZIP) [file pone.0316842.s008.zip › Raw data/Risk of bias summary.pdf]

	Random sequence generation (selection bias)	Allocation concealment (selection bias)	Blinding of participants and personnel (performance bias)	Blinding of outcome assessment (detection bias)	Incomplete outcome data (attrition bias)	Selective reporting (reporting bias)	Other bias
Afsane Ahmadi 2020	+	?	+	?	+	+	?
Christopher Lum 2007	+	+	+	?	+	+	+
Claire de Bisschop 2021	+	?	+	?	+	+	?
Elham PIRABBASI 2016	+	+	−	?	+	+	?
Fares Gouzi 2019	+	?	+	?	+	+	+
Miek Hornikx 2012	+	+	+	?	+	+	+
Peter Santer 2020	+	+	+	?	+	+	?
Philip C. Calder 2017	+	?	+	?	+	+	+
R.W. Dal Negro 2010	+	?	?	?	+	?	?
R.W. Dal Negro 2012	+	+	?	+	+	?	?
Rachida Rafiq 2017	+	+	+	+	+	?	?
Takashi Ogasawara 2018	+	?	?	+	+	+	?
